# Two calmodulin binding elements contribute distinctly to TRPA1 calcium desensitization

**DOI:** 10.1016/j.jbc.2025.111044

**Published:** 2025-12-13

**Authors:** Gregory Quevedo, Kehinde M. Taiwo, Justin H. Sanders, Glory A. Adekanye, Candice E. Paulsen

**Affiliations:** 1 Department of Molecular Biophysics and Biochemistry, Yale University, New Haven, Connecticut, USA; 2 Wu Tsai Institute, Yale University, New Haven, Connecticut, USA

**Keywords:** ion channel, TRP channel, calmodulin, calcium regulation, biochemistry, biophysics

## Abstract

The “wasabi receptor,” transient receptor potential ankyrin 1 (TRPA1), is a critical pain receptor that is activated by reactive environmental and endogenous irritants to initiate pain perception, local inflammation, and protective behaviors. TRPA1 is a calcium-permeable ion channel; calcium influx tightly controls channel function by first potentiating currents and then triggering rapid desensitization. Here, we provide evidence that the universal calcium sensor calmodulin (CaM) controls TRPA1 desensitization by engaging two distinct CaM-binding sites in the cytoplasmic C terminus: the distal C-terminal CaM binding element (DCTCaMBE) and the TRP CaMBD. Specifically, we found that CaM binds TRPA1 at the DCTCaMBE beginning at low calcium and the TRP CaMBD at high calcium concentrations. Pull-down experiments revealed that the DCTCaMBE and TRP CaMBD exhibit distinct CaM lobe binding specificities. Competition experiments showed that CaM can bind both sites simultaneously as isolated peptides. Molecular modeling identified residues predicted to contribute to the CaM–TRP CaMBD interaction. Mutation of these residues revealed that CaM binding to the TRP CaMBD controls a second kinetic step in TRPA1 desensitization. Finally, complete ablation of CaM binding at both the TRPA1 DCTCaMBE and TRP CaMBD showed that they contribute to a concerted desensitization mechanism. Together, these results support a model where CaM associates with TRPA1 at rest through the DCTCaMBE, which primes the channel for rapid desensitization. As intracellular calcium levels rise, CaM then binds the TRP CaMBD—through bridged or separate interactions—to promote a terminal desensitization step. Our work provides further mechanistic insight into how calcium and CaM tightly control TRPA1 channel function to promote nociceptive signaling.

The transient receptor potential (TRP) channels are the second largest class of ion channels, consisting of 28 members in mammals (1, 2, 3, 4). A subset of these channels is expressed by sensory neurons in the dorsal root, trigeminal, and nodose ganglia, where they serve as detectors of noxious thermal, mechanical, and/or chemical stimuli (5, 6, 7). Of these, TRP ankyrin 1 (TRPA1) is the chief chemosensor that allows us to detect harmful environmental and endogenous chemical irritants (8, 9, 10). TRPA1 activation by these chemicals initiates pain signaling, local inflammation, and protective behaviors in humans and rodents (9, 11, 12, 13). Many of these irritants—including allyl isothiocyanate (AITC) from wasabi and mustard, cinnamaldehyde from cinnamon, acrolein from burning vegetation, and 4-hydroxynonenal produced endogenously during inflammation—are reactive chemicals that activate TRPA1 by covalently modifying three key, conserved cysteine residues in the cytoplasmic N terminus (Fig. 1, black) (8, 9, 10, 14, 15, 16). The resulting modifications can persist for more than 1 h, which produces sustained TRPA1 activation in the absence of calcium (Ca^2+^) (15, 17).

**Figure 1:**
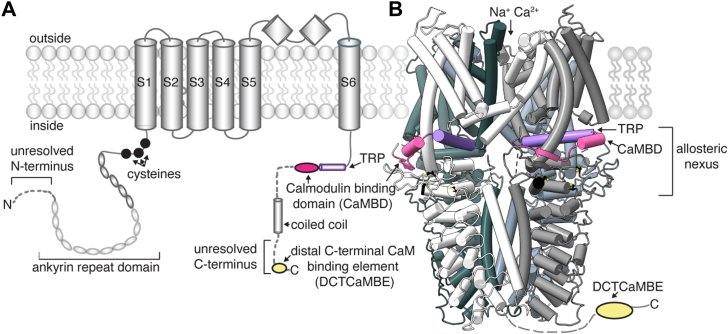
**TRPA1 has two structurally distinct putative CaM binding elements**. *A*, cartoon schematic of a full-length hTRPA1 monomeric subunit. Three conserved cysteine residues involved in electrophile agonist activation (*black*), the TRP helix (*purple*), the previously identified calmodulin-binding domain (CaMBD, *pink*) and distal C-terminal calmodulin binding element (DCTCaMBE, *yellow*) are denoted. *Dashes* and *transparencies* indicate unresolved regions in cryo-EM structures. *B*, *Ribbon diagram* of WT hTRPA1 atomic model for residues K447-E1079 from the homotetrameric channel (PDB: 6V9W). Subunits distinguished by color. The cysteines, CaMBD, and TRP helix are colored as in (*A*). The DCTCaMBE (*yellow oval*) and the structurally unresolved C termini are diagramed extending from the coiled coil. *Dashed line* indicates unknown structural placement. *Bracket* denotes the allosteric nexus. CaM, calmodulin; hTRPA1, human TRPA1; TRP, transient receptor potential; TRPA1, transient receptor potential ankyrin 1.

TRPA1 is a homotetrameric Ca^2+^-permeable nonselective cation channel (Fig. 1) (8, 10, 18, 19). Each subunit has a large cytoplasmic N-terminal domain containing an ankyrin repeat (AR) domain that houses 16 ARs—which gives TRPA1 its namesake—and an “allosteric nexus” with the key cysteines targeted by reactive irritants (Fig. 1). The transmembrane domain (TMD) consists of six transmembrane helices (S1–S6) where S1-S4 form the voltage-sensor like domain and the ion conduction pathway is lined by S5, S6, and the two intervening pore helices (Fig. 1). The voltage-sensor like domain and pore are connected through the S4-S5 linker and tetramers assemble in a domain-swapped configuration within the TMD, like most TRP channels and potassium channels, which promotes concerted channel gating (Fig. 1) (4, 18). The cytoplasmic C terminus contains a TRP helix conserved in all TRP channels, a coiled-coil domain, and two calmodulin (CaM) binding elements in the membrane proximal and membrane distal regions—the CaM-binding domain (CaMBD) and the distal C-terminal CaM binding element (DCTCaMBE), respectively (Fig. 1, purple, pink, and yellow) (18, 20, 21). To date, only 50% of the human TRPA1 (hTRPA1) sequence is ordered in cryo-EM structures (Fig. 1, dashes and transparencies denote unresolved elements) (18, 22, 23). Nonetheless, structures of hTRPA1 in the closed and open states reveal that the allosteric nexus, TMD, TRP helix, and CaMBD undergo large conformational changes during gating (23).

As a Ca^2+^ permeable channel, TRPA1 activity is tightly regulated to avoid spurious Ca^2+^ signaling and cytotoxicity. Mammalian TRPA1 exhibits tight Ca^2+^ regulation characterized by potentiation upon initial Ca^2+^ influx and rapid desensitization as intracellular Ca^2+^ levels rise (10, 24, 25). This important regulatory mechanism serves to restrict channel function, limit Ca^2+^ signaling, and promote TRPA1 utility as an acute pain sensor amidst prolonged chemical modification by reactive irritants. Despite this importance, the mechanisms governing TRPA1 Ca^2+^ regulation have remained unclear. We recently identified the DCTCaMBE in the hTRPA1 structurally unresolved cytoplasmic C terminus (Fig. 1, yellow oval) (21). CaM binding at the DCTCaMBE is critical for rapid TRPA1 desensitization but it is not required for potentiation, providing evidence that these are separate regulatory steps (21). The hTRPA1 DCTCaMBE resides in the final 18 amino acids of the protein sequence within a cytoplasmic, 40 amino acid–long structurally unresolved element beginning ∼80 Å below the channel pore (Fig. 1). It is currently unclear how CaM engagement at a site distal from the pore promotes rapid desensitization.

CaM is a highly conserved ∼17 kDa soluble protein with N- and C-lobes connected by a flexible linker; each lobe contains two EF hand motifs that allow CaM to bind up to four Ca^2+^ ions (26, 27). In isolation, the EF hands in the CaM C-lobe (EF hands 3 and 4) have a 10-fold higher affinity for Ca^2+^ than those in the N-lobe (EF hands 1 and 2) (28, 29). Ca^2+^ binding to the EF hands induces conformational changes that expose hydrophobic patches in the lobes that bind amphipathic helices in effector proteins (26, 27). Because the N- and C-lobes exhibit distinct Ca^2+^ sensitivities, CaM communicates Ca^2+^ signals to hundreds of effector proteins, including many ion channels, by sequential engagement of these lobes to initiate conformational changes that affect protein function (27, 30, 31, 32). We previously found that the hTRPA1 DCTCaMBE engages CaM exclusively through the C-lobe at basal Ca^2+^ concentrations, which allows hTRPA1 and CaM to pre-associate in cells at rest (21). We and others have also found that hTRPA1 regulation by CaM only requires a functional C-lobe (20, 21). It is currently unknown whether the CaM N-lobe also contributes to channel regulation.

Here, we tested the hypothesis that CaM controls hTRPA1 desensitization by also engaging the pore-proximal CaMBD, where binding is expected to influence channel function—either through a bridged interaction with the DCTCaMBE or through separate binding events (Fig. 1, pink) (20). Using CaM-agarose binding assays, ratiometric Ca^2+^ imaging, molecular modeling, two-electrode voltage clamp (TEVC) electrophysiology, and pull-down experiments, we find that (1) CaM engages the DCTCaMBE across all Ca^2+^ concentrations beginning at basal Ca^2+^ and both the TRP helix and the CaMBD at high Ca^2+^ levels fully accounting for TRPA1 CaM binding across all physiological Ca^2+^ concentrations, (2) CaM binding at both sites contributes to a concerted desensitization mechanism, (3) the DCTCaMBE and TRP CaMBD exhibit distinct CaM lobe binding specificities, and (4) CaM can engage both binding sites simultaneously as isolated peptides. Together, our results support a model where CaM first binds hTRPA1 at the DCTCaMBE with its C-lobe priming the channel to initiate rapid desensitization upon Ca^2+^ influx. CaM then engages the TRP CaMBD, possibly through a bridged interaction, *via* the N-lobe as intracellular Ca^2+^ levels rise to affect a terminal step in desensitization. Such two-site regulatory mechanisms and bridged interactions have been proposed for CaM- and Ca^2+^-mediated small-conductance potassium (SK) channel activation, TRPV5 and TRPV6 inhibition, as well as regulation of voltage-gated ion channels (30, 32, 33, 34, 35, 36, 37, 38, 39, 40, 41). Future structural studies are needed to discern whether this two-site regulatory mechanism occurs through a bridged interaction or separate CaM binding events.

## Results

### The TRP helix contributes to CaM binding at the TRPA1 CaMBD

**Figure 2:**
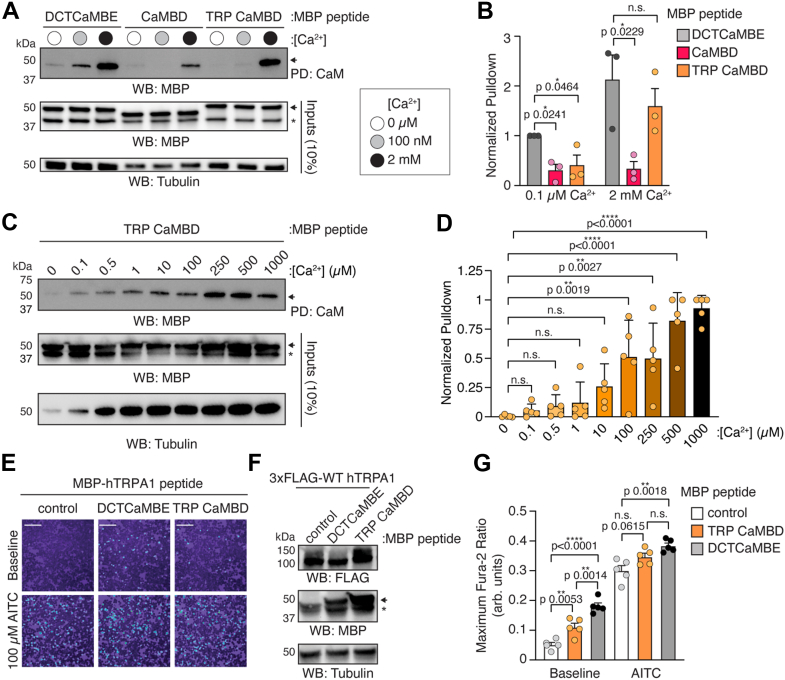
**The TRP CaMBD binds CaM and can influence TRPA1 function**. *A*, immunoblotting analysis of the indicated MBP-hTRPA1 peptide constructs after CaM-agarose pulldown at 0 μM (*white circle*), 100 nM (*gray circle*), or 2 mM (*black circle*) Ca^2+^ concentrations from lysates of HEK293T cells transfected with MBP-DCTCaMBE (hTRPA1^1089-1119^), MBP-CaMBD (hTRPA1^991-1008^), or MBP-TRP CaMBD (hTRPA1^971-1008^) peptides. Blot is representative of three independent experiments. Samples were probed using an anti-MBP primary antibody and an HRP-conjugated anti-mouse secondary antibody. Tubulin from whole-cell lysates (10%, inputs) was the loading control. *B*, quantification of CaM-agarose pulldowns represented in (A) from cells expressing MBP-DCTCaMBE (*gray*), CaMBD (*pink*), or TRP CaMBD (*orange*) hTRPA1 peptides. Pulldown was normalized to the DCTCaMBE peptide at 0.1 μM Ca^2+^ average. Data represent mean ± SD. n = 3 independent experiments, one-way ANOVA (0.1 μM Ca^2+^: F = 7.355, *p* = 0.0243; 2 mM Ca^2+^: F = 6.425, *p* = 0.0323) with Bonferroni’s *post hoc* analysis. *C*, immunoblotting analysis of the MBP-TRP CaMBD peptide after CaM-agarose pulldown at the indicated Ca^2+^ concentrations. Samples were probed as in (*A*). Blot is representative of five independent experiments. *D*, quantification of CaM-agarose pulldowns represented in (*C*). Pulldown was normalized to the maximum binding event within each biological replicate. Data represent mean ± SD. n = 5 independent experiments, one-way ANOVA (F = 5.26, *p* < 0.0001) with Bonferroni’s *post hoc* analysis. *E*, ratiometric calcium imaging of HEK293T cells cotransfected with WT hTRPA1 and free MBP (control), MBP-DCTCaMBE, or MBP-TRP CaMBD. Cells were stimulated with 100 μM AITC. Images are representative of five independent experiments. Scale bars represent 100 μm. *F*, representative immunoblotting analysis of the cells used for calcium imaging in (*E*). Samples were probed using an HRP-conjugated anti-FLAG antibody. Tubulin was the loading control. Blot is representative of five independent experiments. *A*, *C*, and *E*, *asterisk* (∗) denotes free MBP. *Arrow* denotes MBP-tagged peptide. *G*, quantification of baseline and 100 μM AITC-evoked change in Fura-2 ratio of data from panel (*E*). Data represent mean ± SD. n = 5 independent experiments, one-way ANOVA (baseline: F = 37.14, *p* < 0.0001; AITC: F = 10.34, *p* = 0.0025) with Tukey’s *post hoc* analysis. *B*, *D*, and *G p* values indicated in figure panels. AITC, allyl isothiocyanate; CaM, calmodulin; CaMBD, calmodulin-binding domain; DCTCaMBE, distal C-terminal CaM binding element; HEK, human embryonic kidney cells; hTRPA1, human TRPA1; TRP, transient receptor potential; TRPA1, transient receptor potential ankyrin 1.

We recently identified a high affinity, highly conserved CaM binding element in the hTRPA1 distal cytoplasmic C terminus (Fig. 1; the DCTCaMBE) (21). Though this region is structurally unresolved, the DCTCaMBE is predicted to form an α-helix that binds CaM through a set of hydrophobic and basic residues (21). An MBP-tagged TRPA1 C-terminal peptide containing the DCTCaMBE (hTRPA1^1089-1119^) retains the ability to bind CaM in a Ca^2+^-dependent manner at both basal and extracellular Ca^2+^ concentrations (Fig. 2, *A* and *B*, 100 nM and 2 mM Ca^2+^, respectively). To initially ask whether the CaMBD also contributes to TRPA1 CaM engagement, we similarly tested an MBP-tagged CaMBD peptide (hTRPA1^991-1008^) in CaM-agarose binding assays (Fig. 2, *A* and *B*, CaMBD). The CaMBD peptide bound CaM only in the presence of 2 mM Ca^2+^ (Fig. 2, *A* and *B*, pink). Notably, the amount of bound CaMBD peptide was significantly lower than the DCTCaMBE peptide, suggesting that this is a low affinity interaction (Fig. 2, *A* and *B*, compare gray and pink bars).

The CaMBD is formed by an elbow helix and a β-strand; CaM-binding motifs are generally composed of one or two α-helices (Fig. 1*B*) (20, 37, 40, 42). Notably, these atypical structural features are unlikely to fold correctly as an isolated peptide without the full channel context, which could contribute to the lower binding capability. In the TRPA1 structure, the CaMBD elbow helix is preceded by the TRP helix, which contributes to TRP channel gating and may promote formation of the elbow helix in an isolated peptide (Fig. 1, purple) (23). Therefore, we next asked whether a peptide also containing the TRP helix would exhibit improved CaM-binding properties. We found that this TRP CaMBD peptide (hTRPA1^971-1008^) exhibited more CaM binding than the CaMBD alone and that it demonstrated comparable binding to the DCTCaMBE peptide at 2 mM Ca^2+^, suggesting that the TRP helix may promote or contribute to CaM engagement (Fig. 2, *A* and *B*, orange).

These initial assays suggest that the DCTCaMBE and TRP CaMBD peptides bind CaM with distinct Ca^2+^ sensitivities. To determine the lowest Ca^2+^ concentration at which the TRP CaMBD peptide binds CaM, we performed assays with a wide range of physiologically relevant Ca^2+^ concentrations spanning basal intracellular (0.1 μM) to extracellular (mM) levels. These assays confirmed that the TRP CaMBD peptide binds CaM in a Ca^2+^-dependent manner with significant binding at Ca^2+^ concentrations above 100 μM (Fig. 2, *C* and *D*). Interestingly, extracellular Ca^2+^ concentrations above 500 μM are required to trigger appreciable TRPA1 desensitization suggesting that CaM may bind the TRP CaMBD upon sufficient Ca^2+^ influx through open TRPA1 channels (20, 21, 25).

The DCTCaMBE peptide binds CaM at nanomolar affinity and competes with WT hTRPA1 for CaM binding when coexpressed in HEK293T cells (21). By displacing CaM from WT hTRPA1, the DCTCaMBE peptide yields hyperactive channels in ratiometric Ca^2+^ imaging assays both in the absence and presence of agonist (Fig. 2, *E*–*G*, compare white and gray bars) (21). To determine whether the TRP CaMBD also affects WT hTRPA1 activity, we coexpressed the TRP CaMBD peptide with WT hTRPA1 in HEK293T cells. Coexpression with the TRP CaMBD significantly enhanced basal channel activity, albeit to a lower extent than the DCTCaMBE peptide, and is trending toward enhancing agonist-evoked activity, suggesting that it may also compete with WT hTRPA1 for CaM binding (Fig. 2, *E*–*G*, compare white and orange bars). Indeed, a CaMBD peptide was previously shown to compete with full-length TRPA1 for CaM binding and to affect channel function (20). The lower effect of the TRP CaMBD peptide than the DCTCaMBE peptide on influencing WT hTRPA1 function is consistent with the TRP CaMBD peptide binding CaM with a lower affinity and/or requiring a higher Ca^2+^ concentration than the DCTCaMBE peptide. Our peptide binding assays above show that the TRP CaMBD peptide binds CaM across physiologically relevant Ca^2+^ concentrations with significant binding only observed above 100 μM Ca^2+^ (Fig. 2, *C* and *D*). Importantly, prior single channel recordings have revealed that TRPA1 channels are flickery in the absence of agonist (15, 24, 25, 43, 44, 45), which seemingly brings in enough Ca^2+^ to allow the TRP CaMBD peptide to influence baseline channel function in ratiometric Ca^2+^ imaging experiments (Fig. 2, *E* and *G*). Altogether, these findings suggest that the addition of the TRP helix enhances the ability of the CaMBD to bind CaM and that the TRP CaMBD may contribute to TRPA1 Ca^2+^ regulation albeit to a lesser extent than the DCTCaMBE. Moreover, the distinct Ca^2+^ concentration dependencies for the DCTCaMBE and TRP CaMBD to bind CaM hint that they may bind distinct CaM lobes (27). Moving forward, all peptide-based experiments were performed with the TRP CaMBD.

### Modeling predicts key residues for CaM binding to the TRPA1 DCTCaMBE and TRP CaMBD

**Figure 3:**
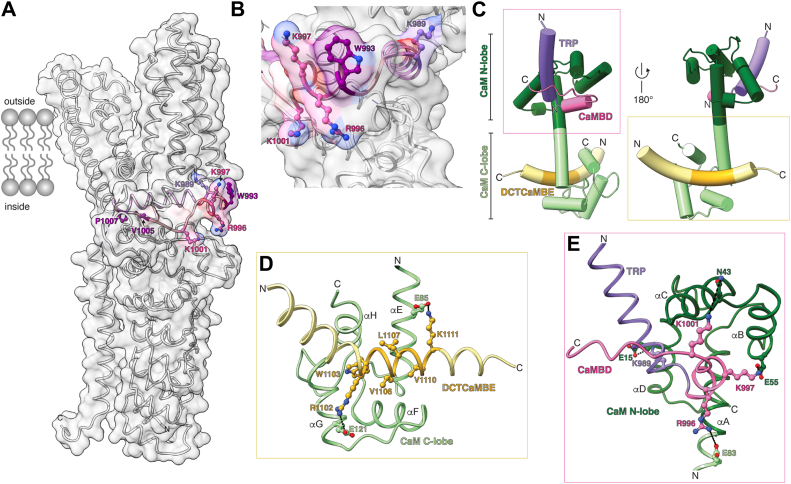
**AlphaFold2-multimer predicts CaM engages the DCTCaMBE and the TRP CaMBD through distinct lobes**. *A*, *ribbon diagram* of a single WT hTRPA1 subunit atomic model for residues K447-E1079 (PDB: 6V9W) overlaid with surface density (*white transparent*). The TRP helix (*purple*, residues 976–990) and CaMBD (*pink*, residues 991–1010) are denoted. CaMBD residues previously identified (*pink*) are depicted as *balls* and *sticks*. V1005 and P1007 are buried in the subunit structure. W993 is surface exposed. TRP (*purple*) and CaMBD (*pink*) residues identified in this study are depicted as *balls* and *sticks*. These residues—K989, R996, K997, and K1001—are surface exposed. *B*, zoom in on surface exposed TRP CaMBD residues from panel (*A*). *C*, *ribbon diagram* of hCaM atomic model (*green*) in complex with the hTRPA1 TRP helix (*purple*, residues 976–990), the CaMBD (*pink*, residues 991–1010), and the TRPA1 disordered C terminus (*yellow*, residues 1089–1119) including the DCTCaMBE (*golden rod*, residues 1102–1111) as predicted by AlphaFold2. *D*, *ribbon diagram* of CaM C-lobe and the DCTCaMBE from panel (*C*) with residues previously identified mediating hydrophobic and polar interactions depicted as *balls* and *sticks*. *E*, *ribbon diagram* of CaM N-lobe and the TRP-CaMBD from panel (*C*) identified here with residues mediating polar interactions depicted as balls and sticks. CaM, calmodulin; CaMBD, calmodulin-binding domain; DCTCaMBE, distal C-terminal CaM binding element; hTRPA1, human TRPA1; TRP, transient receptor potential; TRPA1, transient receptor potential ankyrin 1.

CaMBD residues W993, V1005, and P1007 (mouse numbering: W996, V1008, P1010) were previously proposed to contribute to the TRPA1–CaM interaction since individual mutation of these residues reduced CaM binding of a CaMBD peptide and perturbed TRPA1 Ca^2+^ regulation (20). Notably, V1005 and P1007 are buried in the hTRPA1 fold where they may stabilize the channel structure and would be unable to engage CaM without a significant structural rearrangement (Fig. 3*A*). In contrast, W993 is solvent accessible and could contribute to CaM binding (Fig. 3, *A* and *B*), however, mutation of this residue only reduced CaM binding by approximately half in a CaMBD peptide suggesting that other residues are required for CaM engagement (see ref (20)).

We previously used molecular modeling to identify key DCTCaMBE residues that contribute to CaM binding and TRPA1 Ca^2+^ regulation (21). Moreover, our prior work showed that the TRPA1 DCTCaMBE binds CaM best at basal Ca^2+^ concentration and that this interaction is mediated exclusively with the CaM C-lobe (21). Because the TRP CaMBD peptide only binds CaM above 100 μM Ca^2+^, this raises the intriguing possibility that the TRP CaMBD may engage the CaM N-lobe. Thus, we generated an AlphaFold2-multimer model to predict whether and how CaM could engage the DCTCaMBE and the TRP CaMBD (Figs. 3*C* and S1). This model predicts that the CaM C-lobe engages the DCTCaMBE as an α-helix, while the CaM N-lobe engages the TRP CaMBD through the TRP helix and the CaMBD elbow helix (Fig. 3*C*). To initially validate this model, we confirmed that it predicts that the DCTCaMBE engages CaM through the six key residues we identified previously (Fig. 3*D*, ball and stick residues) (21). Thus, despite the lower confidence score for the TRP CaMBD than that for the DCTCaMBE, we decided to also use our model to predict TRP CaMBD residues that might contribute to CaM engagement (Fig. S1*A*). Our model predicts that K989 in the TRP helix and K997 and K1001 from the CaMBD elbow helix form salt bridges or H-bond interactions with E15, E55, or N43 from the CaM N-lobe, respectively (Fig. 3*E*). Moreover, our model predicts that R996 of the CaMBD elbow helix forms a salt bridge with E83 in the linker between the CaM N- and C-lobes (Fig. 3*E*). Each of these predicted residues are solvent accessible in the TRPA1 structure (Fig. 3, *A* and *B*), suggesting that they could contribute to TRPA1-CaM binding and TRPA1 Ca^2+^ regulation.

### TRPA1 engages CaM through the DCTCaMBE and TRP CaMBD

**Figure 4:**
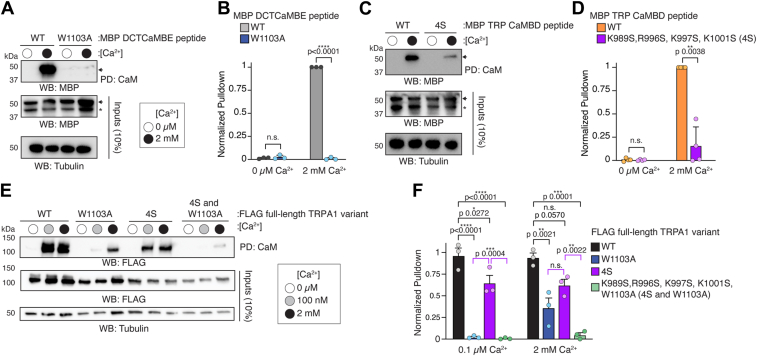
**TRPA1 engages CaM through two distinct sites across all calcium concentrations**. *A*, immunoblotting analysis of WT or the W1103A mutant MBP-DCTCaMBE peptide after CaM-agarose pulldown at 0 μM (*white circle*) or 2 mM (*black circle*) Ca^2+^ concentrations from lysates of HEK293T cells transfected with the indicated peptides. Blot is representative of three independent experiments. Samples were probed as in Figure 2*A*. *B*, quantification of CaM-agarose pulldowns represented in (*A*). Pulldown was normalized to the maximum binding event within each biological replicate. Data represent mean ± SD. n = 3 independent experiments, two-tailed Student’s *t* test. *C*, immunoblotting analysis of WT or the K989S/R996S/K997S/K1001S (4S) mutant MBP-TRP CaMBD peptide after CaM-agarose pulldown at 0 μM (*white circle*) or 2 mM (*black circle*) Ca^2+^ concentrations from lysates of HEK293T cells transfected with the indicated peptides. Blot is representative of four independent experiments. Samples were probed as in Figure 2*A*. *A* and *C*, *asterisk* (∗) denotes free MBP. *Arrow* denotes MBP-tagged peptide. *D*, quantification of CaM-agarose pulldowns represented in (*C*). Pulldown was normalized to the maximum binding event within each biological replicate. Data represent mean ± SD. n = 4 independent experiments, two-tailed Student’s *t* test. *E*, immunoblotting analysis of the indicated 3xFLAG-hTRPA1 constructs after CaM-agarose pulldown at 0 μM (*white circle*), 100 nM (*gray circle*), or 2 mM (*black circle*) Ca^2+^ concentrations from lysates of HEK293T cells transfected with 3xFLAG-WT, W1103A, 4S, or K989S/R996S/K997S/K1001S/W1103A (4S and W1103A) hTRPA1. Blot is representative of three independent experiments. Samples were probed using an HRP-conjugated anti-FLAG antibody. Tubulin from whole-cell lysates (10%, inputs) was the loading control. *F*, quantification of CaM-agarose pulldowns represented in (*E*). Pulldown was normalized to the WT hTRPA1 0.1 μM Ca^2+^ average. Data represent mean ± SD. n = 3 independent experiments, one-way ANOVA (0.1 μM Ca^2+^: F = 59.18, *p* < 0.0001; 2 mM Ca^2+^: F = 27.78, *p* = 0.0001) with Tukey’s *post hoc* analysis. *B*, *D*, and *F*, *p* values indicated in figure panels. AITC, allyl isothiocyanate; CaM, calmodulin; CaMBD, calmodulin-binding domain; DCTCaMBE, distal C-terminal CaM binding element; HEK, human embryonic kidney cells; hTRPA1, human TRPA1; TRP, transient receptor potential; TRPA1, transient receptor potential ankyrin 1.

To initially ask whether residues predicted in our AlphaFold2-multimer model contribute to CaM binding at the DCTCaMBE and TRP CaMBD sites, we performed CaM-agarose binding assays with WT and mutant peptides at Ca^2+^-free (0 mM) and saturating Ca^2+^ concentrations (2 mM). Consistent with our prior work showing that the W1103 hydrophobic anchor is critical to the DCTCaMBE-CaM interaction, the W1103A mutation ablated CaM binding in the isolated DCTCaMBE peptide (Fig. 4, *A* and *B*, blue). In similar experiments, we mutated the four basic residues predicted in our AlphaFold2-multimer model to serine (K989S, R996S, K997S, and K1001S—hereafter referred to as the 4S mutant) in the TRP CaMBD peptide, and we found that the 4S mutations significantly reduced CaM binding by 80% compared to the WT TRP CaMBD peptide (Fig. 4, *C* and *D*, purple). These data suggest that these residues are important for a TRPA1–CaM interaction through the TRP CaMBD.

Next, we tested the contribution of the DCTCaMBE and TRP CaMBD residues to CaM binding in the full-length channel. Consistent with our prior work, we found that the W1103A mutant lost CaM binding at 100 nM Ca^2+^, however, some residual binding is observed with W1103A hTRPA1 at 2 mM Ca^2+^ (Fig. 4, *E* and *F*, blue) (21). Since the W1103A mutant ablated CaM binding in the DCTCaMBE peptide at 2 mM Ca^2+^ (Fig. 4, *A* and *B*, blue), this residual binding may be due to engagement at the TRP CaMBD at the higher Ca^2+^ concentration. The 4S hTRPA1 mutant exhibited slightly reduced CaM binding at 100 nM and 2 mM Ca^2+^ concentrations (Fig. 4, *E* and *F*, purple). Interestingly, there was no significant difference in CaM binding at 2 mM Ca^2+^ between the W1103A or 4S mutants suggesting that affecting CaM binding at the DCTCaMBE or the TRP CaMBD similarly impacts CaM engagement at heightened Ca^2+^ concentrations in the full-length channel (Fig. 4, *E* and *F*, compare blue and purple). In a combination mutant harboring the W1103A and the 4S mutations, we observed a nearly complete loss in CaM binding across all Ca^2+^ concentrations (Fig. 4, *E* and *F*, green).

These collective findings suggest that the DCTCaMBE and TRP CaMBD are both CaM binding elements in the full-length channel and that the membrane proximal TRP CaMBD contributes to CaM binding at higher Ca^2+^ concentrations following Ca^2+^ influx through open TRPA1 channels. They also hint that the ability to bind CaM at the membrane proximal TRP CaMBD in the full-length channel may act secondary to or be influenced by CaM engagement at the distal DCTCaMBE (Fig. 4, *B* and *F*, blue).

### The DCTCaMBE and TRP CaMBD contribute distinctly to TRPA1 Ca^2+^ regulation

**Figure 5:**
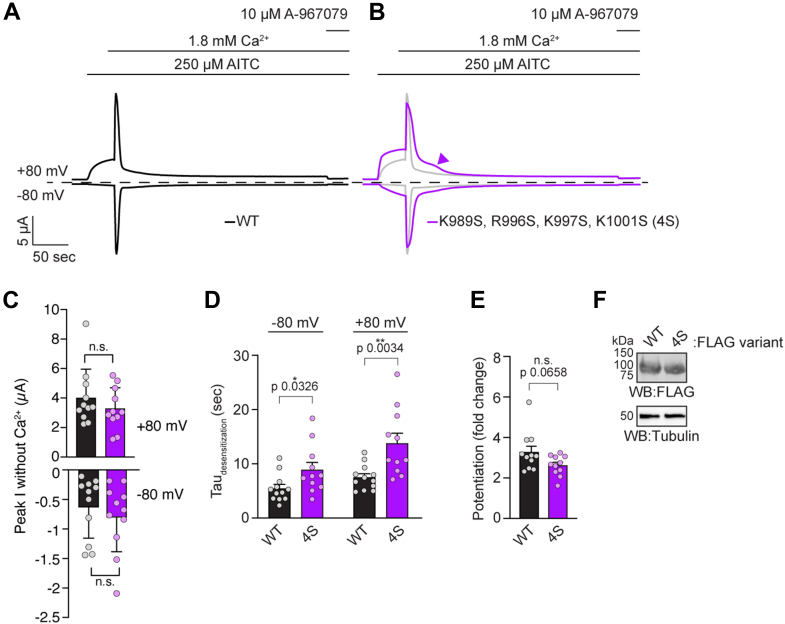
**Perturbation of CaM binding to the TRP CaMBD affects TRPA1 desensitization**. *A–B*, representative time traces at −80 and + 80 mV holding potentials from oocytes expressing WT (*A*, *black*) or K989S/R996S/K997S/K1001S (4S) (*B*, *purple*) hTRPA1. Current evoked with 250 μM AITC in the absence and presence of 1.8 mM extracellular Ca^2+^. Channels were blocked with the hTRPA1 antagonist 10 μM A-967079. *Dashed line* denotes 0 μA current. Protocol of condition application indicated above. WT hTRPA1 trace from (*A*) replotted in (*B*) as transparent trace. *C*, quantification of peak current amplitudes at +80 (*above*) and −80 mV (*below*) evoked by 250 μM AITC in the absence of extracellular Ca^2+^. Colors as in (*A* and *B*). Data represent mean ± SD. n = 11 (4S) or 12 (WT) independent oocytes. *D*, calculated time constants of desensitization from fitting data as in (*A*) and (*B*) to a single-exponential function. Data represent mean ± SD. n = 11 (4S) or 12 (WT) independent oocytes. *E*, calculated fold change potentiation calculated from maximum currents with extracellular Ca^2+^ compared to the maximum currents before Ca^2+^ addition from data as in (*A*) and (*B*) at +80 mV holding potential. Data represent mean ± SD. n = 11 oocytes per condition. *F*, Western blot of lysates from oocytes used for recordings in (*A*) and (*B*) expressing 3xFLAG-tagged hTRPA1 variants, probed using HRP-conjugated anti-FLAG antibody. Tubulin from whole-cell lysates was the loading control. *C-E*, two-tailed Student’s *t* test. *p* values indicated in figure panels. AITC, allyl isothiocyanate; CaM, calmodulin; CaMBD, calmodulin-binding domain; DCTCaMBE, distal C-terminal CaM binding element; hTRPA1, human TRPA1; TRP, transient receptor potential; TRPA1, transient receptor potential ankyrin 1.

We next wanted to understand the contribution that each CaM-binding site plays in TRPA1 Ca^2+^ regulation. First, we expressed WT or 4S hTRPA1 in *Xenopus laevis* oocytes and recorded AITC-evoked currents by TEVC electrophysiology in the absence and then presence of 1.8 mM extracellular Ca^2+^ to observe the basal function, and then potentiation and desensitization phases of channel regulation, as previously reported (Fig. 5) (21, 46, 47). Such experiments revealed that there is no difference in peak current amplitudes evoked by AITC between WT hTRPA1 and the 4S mutant in the absence of extracellular Ca^2+^ (Fig. 5, *A*–*C*). Upon extracellular Ca^2+^ addition, the 4S mutant exhibited robust potentiation and slowed desensitization compared to WT hTRPA1 (Fig. 5, *A* and *B*). We calculated the desensitization rates for WT or 4S hTRPA1 and found that the 4S mutant significantly slowed desensitization 1.6- and 1.8-fold compared to WT hTRPA1 at inward (−80 mV) and outward (+80 mV) currents, respectively (Fig. 5*D*). Moreover, the delayed desensitization in the 4S mutant revealed a second kinetic step in the desensitization pathway that may be specifically affected (Fig. 5*B*, purple arrowhead). To calculate potentiation, we determined the fold change to current amplitudes in the presence of extracellular Ca^2+^ compared to those before Ca^2+^ addition, as reported previously (20, 21, 25). This revealed that the 4S mutant was trending toward reducing potentiation compared to WT hTRPA1, consistent with prior work showing that perturbation of the CaM–CaMBD interaction affects potentiation (Fig. 5*E*) (20). These functional effects of the 4S mutant were not due to altered channel expression (Fig. 5*F*). Despite the 4S TRP CaMBD mutant only partially reducing CaM binding in the full-length channel, it reduced binding by 80% in the isolated peptide suggesting that the 4S mutant perturbs CaM binding at this membrane proximal site in the full-length channel and that residual CaM binding is occurring through the DCTCaMBE (Fig. 4, *C*–*F*). Thus the ∼2-fold slowing to the desensitization rate in this mutant supports a role for CaM binding to the TRP CaMBD in TRPA1 Ca^2+^ regulation. Moreover, this ∼2-fold slowing to the desensitization rate is comparable to the effect previously seen when WT TRPA1 was coexpressed with a CaMBD peptide further supporting a role for CaM binding at this site (20). Nonetheless, it is possible that the 4S mutant impacts channel regulation independent of CaM binding.

**Figure 6:**
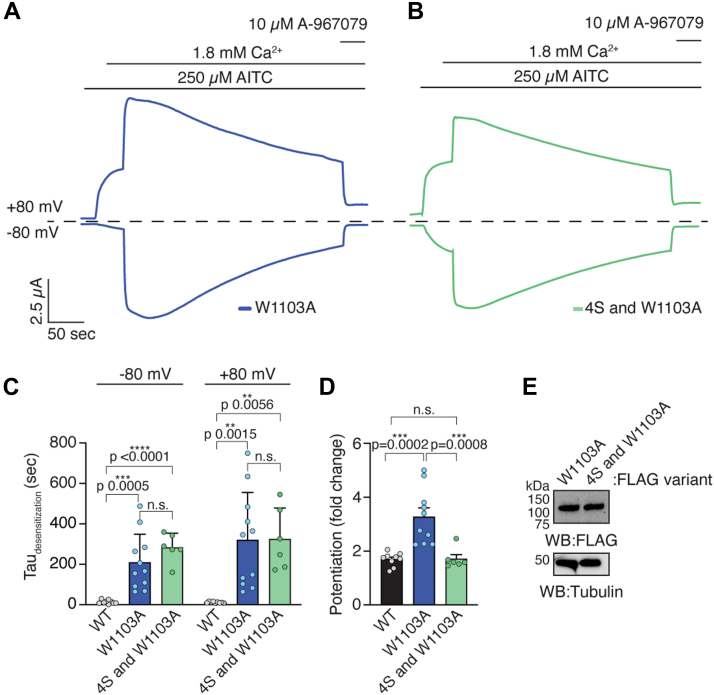
**DCTCaMBE and TRP CaMBD may contribute to the same desensitization mechanism**. *A–B*, representative time traces at −80 and + 80 mV holding potentials from oocytes expressing W1103A (*A*, *blue*) or K989S/R996S/K997S/K1001S/W1103A (*B*, 4S and W1103A, *green*) hTRPA1. Current evoked with 250 μM AITC in the absence and presence of 1.8 mM extracellular Ca^2+^. Channels were blocked with 10 μM A-967079. *Dashed line* denotes 0 μA current. Protocol of condition application indicated above. *C*, calculated time constants of desensitization from fitting data as in (*A*) and (*B*) to a single-exponential function. Data represent mean ± SD. n = 11 (WT), 10 (W1103A), or 6 (4S and W1103A) independent oocytes. *D*, calculated fold change potentiation calculated from maximum currents with extracellular Ca^2+^ compared to the maximum currents before Ca^2+^ addition from data as in (*A*) and (*B*) at +80 mV holding potential. Data represent mean ± SD. n = 9 (WT), 10 (W1103A), or 6 (4S and W1103A) oocytes per condition. *E*, Western blot of lysates from oocytes used for recordings in (*A*) and (*B*) expressing 3xFLAG-tagged hTRPA1 variants, probed as in Figure 5*E*. *C* and *D*, one-way ANOVA (*C*, −80 mV: F = 16.2, *p* < 0.0001; *C*, +80 mV: F = 9.68, *p* = 0.0009; *D*: F = 15.19, *p* < 0.0001) with Tukey’s *post hoc* analysis. *p* values indicated in figure panels. AITC, allyl isothiocyanate; CaM, calmodulin; CaMBD, calmodulin-binding domain; DCTCaMBE, distal C-terminal CaM binding element; hTRPA1, human TRPA1; TRP, transient receptor potential; TRPA1, transient receptor potential ankyrin 1.

Consistent with our prior work, the W1103A hTRPA1 mutant significantly slowed desensitization 15.5- and 28-fold compared to WT hTRPA1 in the inward and outward directions, respectively (Fig. 6, *A* and *C*, blue) (21). Notably, while the W1103A hTRPA1 mutant exhibits significantly delayed desensitization, this contrasts with human-rattlesnake chimeric channels that exhibit no desensitization, suggesting that TRPA1 Ca^2+^ regulation can be further perturbed (21, 47). Therefore, we asked whether the combination W1103A and 4S hTRPA1 mutant, which ablates CaM binding across all Ca^2+^ concentrations, would have a greater effect on hTRPA1 Ca^2+^ regulation than the W1103A hTRPA1 mutant alone (Fig. 4, *E* and *F*, green). Nonetheless, introduction of the TRP CaMBD 4S mutant did not significantly further impact desensitization in the inward or outward directions compared to the W1103A hTRPA1 mutant alone (Fig. 6, *B* and *C*, green *versus* blue). We calculated potentiation, as above, and observed a significant increase in potentiation with outward currents for W1103A hTRPA1 as previously reported, but not with the combination 4S and W1103A mutant (Fig. 6*D*) (21). We previously rationalized an impact to potentiation by the W1103A mutant as being due to channels having more time for full potentiation in the absence of rapid desensitization (21). The loss of this enhancement to potentiation with the additional 4S mutations suggests that the TRP CaMBD influences potentiation, as previously proposed, and as hinted at by our data with the 4S mutant alone (Fig. 5*E*, purple) (20). Differences in channel activity were not due to differences in protein expression (Fig. 6*E*).

Collectively, these results show that CaM binding to the membrane distal DCTCaMBE and membrane proximal TRP CaMBD contribute distinctly to hTRPA1 Ca^2+^ regulation. Disruption of CaM binding at the membrane distal DCTCaMBE has a significantly more drastic effect on hTRPA1 desensitization than perturbation to the membrane proximal TRP CaMBD. This supports our previous findings that the DCTCaMBE serves as the primary site for hTRPA1 CaM binding and control of desensitization and further reveals that CaM engagement at the TRP CaMBD plays a supporting role (21).

### The DCTCaMBE and TRP CaMBD peptides display distinct CaM lobe binding specificities

Our AlphaFold2-multimer model predicts that CaM could engage the DCTCaMBE and TRP CaMBD simultaneously through its C- and N-lobes, respectively (Fig. 3*C*). Such bridged interactions are common among CaM regulated ion channels where sequential engagement of the C- and N-lobes confers Ca^2+^ signals to effector channels to influence function (30, 32, 33, 34, 35, 36, 37, 38, 39, 40, 41). For such a bridged interaction to occur, the DCTCaMBE and TRP CaMBD would need to engage distinct CaM lobes.

**Figure 7:**
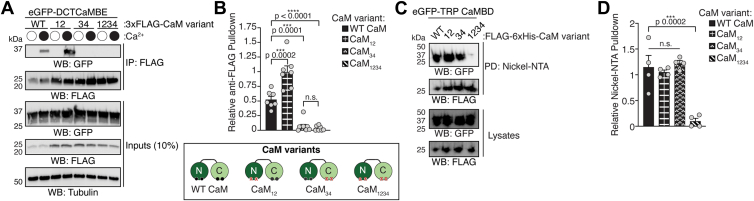
**CaM lobe specificity of both TRPA1 binding elements**. *A*, immunoblotting analysis of GFP-DCTCaMBE peptide (hTRPA1^1089-1119^) with the indicated 3xFLAG-CaM constructs after anti-FLAG pulldowns at 0 (*white circle*) or 2 mM (*black circle*) Ca^2+^ concentrations from lysates of HEK293T cells cotransfected with eGFP-DCTCaMBE and 3xFLAG-WT hCaM, CaM_12_, CaM_34_, or CaM_1234_. Blot is representative of seven independent experiments. Samples were probed using anti-GFP (TRPA1 peptide) and anti-FLAG (CaM) antibodies. Tubulin from whole-cell lysates (10%, inputs) was the loading control. *Bottom box* diagrams CaM variants tested; *red* Xs indicate lobes harboring mutations. *B*, quantification of anti-FLAG pulldowns represented in (*A*). Data represent mean ± SD. n = 7 independent experiments, one-way ANOVA (F = 50, *p* < 0.0001) with Tukey’s *post hoc* analysis. *C*, immunoblotting analysis of eGFP-TRP CaMBD (hTRPA1^971-1008^) copurified from *E*. *coli* with FLAG-6xHis-WT hCaM, CaM_12_, CaM_34_, or CaM_1234_ in the presence of 2 mM Ca^2+^. Nickel-NTA eluates (*top*) and crude cell lysates (*bottom*) were probed using anti-GFP and anti-FLAG antibodies. Experiment is representative of four independent purifications. *D*, quantification of Nickel-NTA pulldowns represented in (*C*). Data represent mean ± SD. n = 4 independent experiments, one-way ANOVA (F = 18.29, *p* < 0.0001) with Bonferroni’s *post hoc* analysis. *B* and *D*, *p* values are indicated in figure panels.CaM, calmodulin; CaMBD, calmodulin-binding domain; DCTCaMBE, distal C-terminal CaM binding element; hTRPA1, human TRPA1; TRP, transient receptor potential; TRPA1, transient receptor potential ankyrin 1.

To experimentally determine which CaM lobes the DCTCaMBE and TRP CaMBD peptides bind, we performed pull-down experiments with a suite of CaM variants containing mutations in the EF hands of the N-lobe (CaM_12_), C-lobe (CaM_34_), or both (CaM_1234_) lobes (Fig. 7*A*; CaM variants) (21). These assays were performed at 0 and 2 mM Ca^2+^ to ensure no or full Ca^2+^ loading of the CaM lobes, respectively. For the DCTCaMBE, an eGFP-tagged peptide was coexpressed with a suite of 3xFLAG-tagged CaM variants in HEK293T cells and complexes were isolated from lysates with anti-FLAG affinity resin. The DCTCaMBE peptide was successfully pulled down by WT CaM and CaM_12_ in a Ca^2+^-dependent manner (Fig. 7, *A* and *B*). Consistent with our prior affinity measurements and 2D-NMR data, the DCTCaMBE peptide associated with the CaM_12_ variant harboring only a functional C-lobe better than WT CaM (Fig. 7, *A* and *B*) (21). In contrast, the DCTCaMBE peptide failed to bind the CaM_34_ or CaM_1234_ variants further supporting that this site engages CaM specifically through the C-lobe (Fig. 7, *A* and *B*).

We observed lower eGFP-TRP CaMBD expression in HEK293T cells, which complicated interpretation of similar anti-FLAG pull-down experiments (*data not shown*). To bypass this, we coexpressed the eGFP-TRP CaMBD peptide with a suite of FLAG-6xHis-CaM variants in *Escherichia coli* and isolated complexes by nickel ion conjugated to nitriloacetic acid pulldown in the presence of 2 mM Ca^2+^ (Fig. 7*C*). Such experiments revealed that the TRP CaMBD peptide bound to WT CaM, CaM_12_, and CaM_34_, but not CaM_1234_, suggesting that the TRP CaMBD binds CaM in a Ca^2+^-dependent manner—since it did not bind CaM_1234_—and that it can bind both the N- and C-lobes (Fig. 7, *C* and *D*).

To further probe the Ca^2+^ dependence of this interaction, the purified TRP CaMBD–CaM complexes were incubated with 10 mM EGTA to chelate all Ca^2+^. In these experiments, we observed a precipitate form only in the complexes containing the TRP CaMBD peptide (*i*.*e*., WT CaM, CaM_12_, and CaM_34_). When we probed the supernatants and precipitates, we found that the TRP CaMBD peptide precipitated when not complexed with CaM (Fig. S2, lanes 4). Moreover, we have found that unlike the DCTCaMBE peptide, the CaMBD and TRP CaMBD peptides cannot be purified as isolated peptides from bacteria that lack endogenous CaM, which complicates further biophysical characterization of the TRP CaMBD–CaM interaction (*data not shown*) (48). This suggests that the TRP CaMBD peptide is unstable in isolation and may require coproduction with CaM to fold properly. Notably, we also found that the TRP CaMBD precipitated slowest from complexes with CaM_34_ rather than WT CaM or CaM_12_, providing some evidence that the TRP CaMBD may bind the lower Ca^2+^ affinity CaM N-lobe best, which is further supported by our observation above that the TRP CaMBD peptide binds CaM above 100 μM Ca^2+^ (Fig. 2, *C* and *D*). Together, these data support our previous findings that the DCTCaMBE binds CaM through its C-lobe and show that the TRP CaMBD can bind both the CaM N- and C-lobes.

### CaM can bind the TRP CaMBD and DCTCaMBE peptides simultaneously

**Figure 8:**
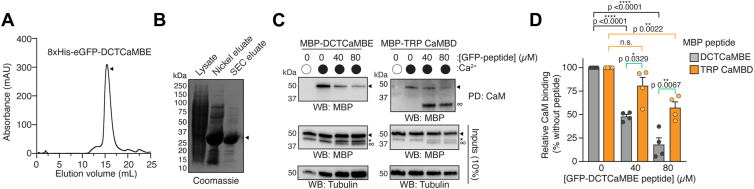
**TRPA1 binding elements may bind CaM simultaneously**. *A* and *B*, purification of 8xHis-eGFP-DCTCaMBE (hTRPA1^1089-1119^) peptide for competition assays. Superdex 200 (*A*) and Coomassie gel (*B*) from the purification are shown. *C*, immunoblotting analysis of MBP-DCTCaMBE (hTRPA1^1089-1119^) or MBP-TRP CaMBD (hTRPA1^971-1008^) after CaM-agarose pulldown at 0 (*white circle*) or 2 mM (*black circle*) Ca^2+^ concentrations in presence of 0, 40, or 80 μM 8xHis-eGFP-DCTCaMBE peptide. Samples were probed as in Figure 2*A*. Blot is representative of four independent experiments. *Asterisk* (∗) denotes free MBP. *Arrow* denotes MBP-tagged peptide. Notably, eGFP fluorescence from the eGFP-DCTCaMBE peptide was detected by the chemiluminescence imaging (infinity symbol, ∞). *D*, quantification of CaM-agarose pulldowns represented in (*C*). Pulldown within each replicate was normalized to MBP-DCTCaMBE (*gray*) or TRP CaMBD (*orange*) at 2 mM Ca^2+^ without 8xHis-eGFP-DCTCaMBE peptide set to 100% binding. Data represent mean ± SD. n = 4 independent experiments, one-way ANOVA (DCTCaMBE: F = 88.68, *p* < 0.0001; TRP CaMBD: F = 11.18, *p* = 0.0036) with Bonferroni’s *post hoc* analysis within each peptide type (*black* and *orange brackets*). Two-tailed Student’s *t* test (*green brackets*) to compare MBP-peptide binding at specific GFP-peptide concentrations. *p* values are indicated in figure panel. CaM, calmodulin; CaMBD, calmodulin-binding domain; DCTCaMBE, distal C-terminal CaM binding element; hTRPA1, human TRPA1; TRP, transient receptor potential; TRPA1, transient receptor potential ankyrin 1.

Since the DCTCaMBE and TRP CaMBD exhibit distinct CaM lobe binding specificities, this raises the possibility that these sites can engage CaM simultaneously as predicted in our AlphaFold2-multimer model (Fig. 3*C*). To test this, we purified an 8xHis-eGFP-DCTCaMBE peptide from bacteria for use in CaM binding competition assays (Fig. 8, *A* and *B*). MBP-tagged DCTCaMBE and TRP CaMBD peptides were expressed separately in HEK293T cells and lysates were used in CaM-agarose pull-down assays in the absence and presence of purified 8xHis-eGFP-DCTCaMBE peptide (Fig. 8, *C* and *D*). As seen above, the MBP-DCTCaMBE and TRP CaMBD peptides bound CaM in a Ca^2+^-dependent manner in the absence of the 8xHis-eGFP-DCTCaMBE peptide (Fig. 8, *C* and *D*). Since the DCTCaMBE only engages the CaM C-lobe, we observed a dose-dependent inhibition of MBP-DCTCaMBE binding to CaM with increasing concentrations of the eGFP-tagged peptide (Fig. 8, *C* and *D*, grey). In contrast, MBP-TRP CaMBD was significantly less affected by the 8xHis-eGFP-DCTCaMBE peptide requiring 80 μM purified peptide to impact binding (Fig. 8, *C* and *D*, orange). The 8xHis-eGFP-DCTCaMBE peptide bound CaM in these assays, ruling out the possibility that the TRP CaMBD prevents DCTCaMBE binding (Fig. 8, *C*, infinity symbol). Together, these results suggest that CaM can engage the DCTCaMBE and TRP CaMBD simultaneously as isolated peptides.

## Discussion

**Figure 9:**
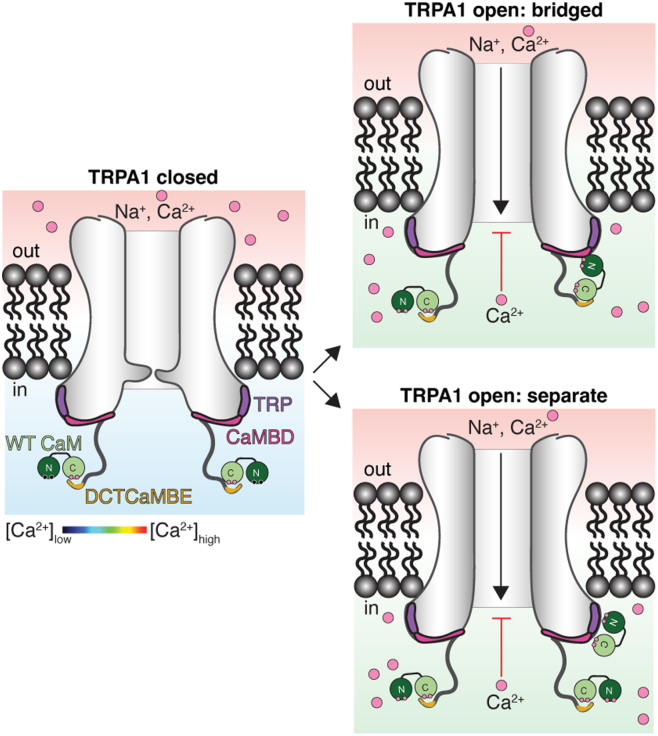
**CaM regulates TRPA1 desensitization by engaging both CaM binding elements**. At rest (*center*), CaM binds the TRPA1 DCTCaMBE through its C-lobe in a Ca^2+^-dependent manner. Upon channel activation, Ca^2+^ influx, and an increase in the local Ca^2+^ concentration (*green color* on the Ca^2+^ concentration heat map), the CaM N-lobe becomes Ca^2+^-loaded and can bind the TRPA1 TRP CaMBD. CaM binding at the TRP CaMBD could be through recruitment of a separate CaM molecule (*bottom*) or by engagement of the N-lobe of a DCTCaMBE-bound CaM molecule through a bridged interaction (*top*). CaM, calmodulin; CaMBD, calmodulin-binding domain; DCTCaMBE, distal C-terminal CaM binding element; hTRPA1, human TRPA1; TRP, transient receptor potential; TRPA1, transient receptor potential ankyrin 1.

Here, we tested the hypothesis that CaM controls hTRPA1 desensitization through two previously identified binding elements—the membrane distal DCTCaMBE and the membrane proximal CaMBD—thereby restricting channel function, limiting Ca^2+^ signaling, and promoting TRPA1 utility as an acute pain sensor (20, 21). Our prior work revealed that CaM binds the DCTCaMBE at basal (100 nM) Ca^2+^ concentration and here we find that CaM binds the CaMBD above 100 μM Ca^2+^ (Fig. 2) (21). Through binding assays and modeling, we show that this interaction is mediated by the TRP helix and an elbow helix at the beginning of the CaMBD (Figs. 2, 3, *C* and *E*). In this model, the CaM N-lobe engages the TRP helix and elbow helix from the side, which is reminiscent of CaM binding to the SK channel (37, 49). Mutation of model-predicted residues in the TRP CaMBD (4S) reduces CaM binding in a TRP CaMBD peptide and the full-length channel and slows desensitization kinetics ∼2-fold compared to WT channels (Figs. 4 and 5). While this impact is modest compared to the 15.5- to 28-fold delay to desensitization rate observed with a DCTCaMBE hTRPA1 mutant (W1103A) (Fig. 6), it is consistent with the effect observed previously by coexpression of WT TRPA1 with a CaMBD peptide (20). The TRP CaMBD mutant may impact a second kinetic phase, and CaM binding at this site may serve as a final step in the desensitization mechanism (Fig. 5). Channels harboring mutations in both the DCTCaMBE and TRP CaMBD, which ablates CaM binding across all Ca^2+^ concentrations, yields no significant additional impact to desensitization rates (Figs. 4 and 6). This suggests that CaM binding at these sites contributes to a concerted desensitization pathway. Finally, we show that the DCTCaMBE only binds the CaM C-lobe while the TRP CaMBD can engage both the CaM N- and C-lobes, and we provide evidence that CaM can engage the DCTCaMBE and TRP CaMBD simultaneously as isolated peptides (Figs. 7 and 8). Together, these results support a model wherein CaM may regulate hTRPA1 desensitization by first pre-associating with the DCTCaMBE at rest and then binding the TRP CaMBD as Ca^2+^ levels rise. This two-site binding could occur through a bridged interaction, as is common among CaM-regulated ion channels (Fig. 9, top) (30, 32, 33, 34, 35, 36, 37, 38, 39, 40, 41), or separate binding events (Fig. 9, bottom). Independent of the specific binding mode, we propose that this two-site engagement facilitates rapid hTRPA1 desensitization upon Ca^2+^ influx.

**Figure 10:**
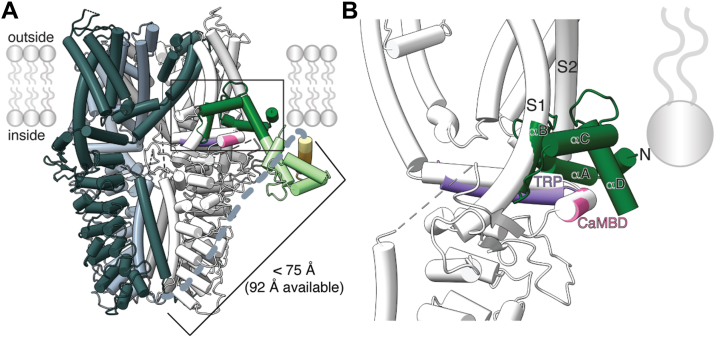
**CaM binding to the TRP CaMBD will require conformational rearrangements**. *A*, AlphaFold2-multimer model from Figure 3*C* docked to the WT hTRPA1 atomic model for residues K447-E1079 from the homotetrameric channel (PDB: 6V9W). Models were aligned by the CaMBD elbow helix in the white subunit (*pink*). *Dashed slate line* denotes connection of E1079 to the DCTCaMBE from the *light slate subunit*. This distance is approximately 75 Å in the structure. The linker can extend 92 Å. *B*, zoom in on region boxed in (*A*) centering on the CaM N-lobe–TRP CaMBD interaction. The CaM alpha helix B clashes with the TRPA1 S1 helix and the CaM N-lobe inserts into the lipid bilayer suggesting that substantial conformational rearrangements are necessary for the interaction to occur. CaM, calmodulin; CaMBD, calmodulin-binding domain; DCTCaMBE, distal C-terminal CaM binding element; hTRPA1, human TRPA1; TRP, transient receptor potential.

For CaM to engage the DCTCaMBE and TRP CaMBD simultaneously through a bridged interaction, the hTRPA1 distal cytoplasmic C terminus would need to extend up toward the pore (Fig. 9, top). The DCTCaMBE and TRP CaMBD are ∼120 amino acids apart in the hTRPA1 primary sequence, and it is currently unknown where the distal C terminus resides in relation to the pore. However, we can use existing structures to predict the feasibility of a bridged interaction. The final resolved residue in the hTRPA1 apo state structure is 74.8 Å away from K989 (TRP helix) and 66.3 Å away from R996 (CaMBD) (Fig. 10*A*). The DCTCaMBE is predicted to bind CaM as an alpha helix formed by 10 residues ending 32 residues into the structurally unresolved distal C terminus. If the preceding 22 residues are fully extended (22 amino acids × 3.5 Å average width per amino acid) and the DCTCaMBE forms an alpha helix (10 amino acids × 1.5 Å pitch/residue in an alpha helix), this region could reach 92 Å (Fig. 10*A*). Thus, there is enough length in the structurally unresolved hTRPA1 distal C terminus to facilitate a bridged interaction with CaM between the DCTCaMBE and TRP CaMBD (Fig. 10*A*). Similar bridged interactions between distinct CaM binding elements in distal and proximal cytoplasmic C-terminal elements have been observed in TRPV5/6, voltage-gated Ca^2+^ channels, and SK channels (34, 37, 40). Future cryo-EM structures of the hTRPA1–CaM complex are needed to discern whether a bridged interaction forms and how it affects channel function.

In the tetrameric hTRPA1 structure, the DCTCaMBE from one subunit would be bridged through CaM to the TRP CaMBD in the opposing subunit, raising the possibility that—if it forms—this interaction would facilitate concerted and efficient desensitization, particularly if each hTRPA1 subunit engages a separate CaM protein (Fig. 10*A*, light slate to white subunits). We currently do not know the stoichiometry of TRPA1 and CaM proteins in the TRPA1–CaM complex. For some oligomeric channels—including ryanodine receptors, TRPC4, and SK channels—each protomer engages a separate CaM protein, whereas in TRPV5 and TRPV6 a single CaM protein binds to the tetrameric channel (34, 37, 40, 50). We previously found that a drastic hTRPA1 truncation mutant which lacks the complete cytoplasmic C terminus including the DCTCaMBE and TRP CaMBD confers gain of function by co-assembling with WT hTRPA1 subunits into hyperactive heteromeric channels (46). Functional channels including heteromers containing at least one truncated subunit exhibited slightly delayed desensitization kinetics hinting that hTRPA1 Ca^2+^ regulation may be fine-tuned by the number of CaM molecules bound. Future work is needed to determine how many CaM proteins engage a homotetrameric hTRPA1 channel to control desensitization.

While our data support a bridged interaction model, we cannot rule out a possibility that the DCTCaMBE and TRP CaMBD engage separate CaM proteins (Fig. 9, bottom). However, we predict that this scenario is less likely than a bridged interaction. Specifically, we previously showed that a minimal TRPA1 construct that yields identical structures to the full-length protein, but which lacks all structurally unresolved elements including the full DCTCaMBE, failed to bind CaM at any Ca^2+^ concentration suggesting that CaM engagement at the TRP CaMBD may require prior association of CaM at the DCTCaMBE to increase the local CaM concentration (Fig. 9) (21, 51). This is easily achieved since the hTRPA1 DCTCaMBE binds CaM best through the C-lobe at basal Ca^2+^ concentration and our prior data support a preassociation of hTRPA1 and CaM through the DCTCaMBE at rest in cells (21).

An intriguing alternate possibility that incorporates both binding mode models is that CaM dissociates from the DCTCaMBE, and CaM binds the TRP CaMBD through both lobes as intracellular Ca^2+^ levels rise. While this binding exchange would require that the DCTCaMBE and TRP CaMBD come in proximity during desensitization, it does not require a bridged interaction *per se*. We previously found that the DCTCaMBE-CaM affinity is weakened from 100 nM to 600 nM when CaM transitions from the C-lobe only Ca^2+^-loaded state to the fully Ca^2+^-loaded state as would occur during channel activation (21). Transfer of CaM from the DCTCaMBE to the TRP CaMBD could occur if the TRP CaMBD-CaM affinity were tighter than 600 nM. Notably, it will be difficult to measure the TRP CaMBD-CaM affinity due to the poor biochemical stability of the TRP CaMBD peptide (Fig. S2), but future TRPA1-CaM costructures could resolve the CaM binding mode.

The proximity of the TRP CaMBD to the hTRPA1 pore predicts that CaM binding at this site will influence channel function (Figs. 1 and 10). Indeed, the TRP helix and underlying allosteric nexus undergo conformational changes during hTRPA1 channel gating; tetrahydrofuran-based antagonists wedge between the TRP helix and the preS1 helix to oppose channel gating, including movements at the S4-S5 linker, demonstrating that this is a highly dynamic region (23, 52). Docking of our AlphaFold2-multimer model to the hTRPA1 apo structure shows that CaM engagement will require substantial conformational rearrangements to accommodate the interaction and to avoid insertion of the CaM N-lobe into the inner leaflet of the membrane bilayer (Fig. 10). Thus, CaM binding at the membrane proximal TRP CaMBD likely induces conformational changes to promote entry into the nonconductive desensitized state, which has not yet been captured structurally.

Collectively, our results support a model where the membrane distal DCTCaMBE is the primary CaM binding site in hTRPA1; CaM binding at this site is critically required for rapid desensitization and CaM binding at the membrane proximal TRP CaMBD plays a secondary role. We propose that CaM engagement through its C-lobe at the DCTCaMBE is also the initial step in a long-range allosteric mechanism that communicates a Ca^2+^ signal from the distal C terminus to the pore. This long-range allosteric mechanism likely involves other structural elements *en route* to the pore. Indeed, an acidic cluster at the base of the coiled coil and a regulatory hinge in the N-terminal ankyrin repeat domain reside between the distal C terminus and the pore in the hTRPA1 structure and these sites have been previously proposed to contribute to desensitization (47, 53). Once the signal propagates to the TRP CaMBD, CaM engagement through its N-lobe at this site facilitates hTRPA1 desensitization by promoting conformational changes that accelerate entry into the desensitized state (Figs. 9 and 10). Consistently, disruption of CaM binding at the TRP CaMBD reveals a slow kinetic phase of desensitization (Fig. 6). While this binding event does not appear to be strictly required for the channel to desensitize—which is consistent with prior observations by us and others that the CaM N-lobe is dispensable for TRPA1 Ca^2+^ regulation—an ∼2-fold slowing to the desensitization rate caused by perturbing this interaction is expected to have a significant physiological effect due to prolonged channel activity (20, 21). Further characterization of the hTRPA1 Ca^2+^ regulatory cycle will reveal how these, and other structural elements, together contribute to the rapid desensitization necessary to functionally restrict this critical pain receptor and the physiological consequences of disrupting this process.

## Experimental procedures

### Plasmid construction

Cloning for 3xFLAG-WT and W1103A hTRPA1, 8xHis-MBP-hTRPA1^1089-1119^ in CMV and bacterial expression vectors, 8xHis-MBP, and the suite of 3xFLAG-CaM variants were reported previously (21, 46). 8xHis-MBP pFastBac1 modified with a CMV promoter hTRPA1^991-1008^ (CaMBD) or TRPA1^971-1008^ (TRP CaMBD) were created by introducing a BamHI site at the −1 position of the codon encoding TRPA1^990^ or TRPA1^970^ by site-directed mutagenesis (Agilent) and digesting the plasmid with BamHI restriction enzyme followed by gel purification. The linearized vector segment was then transformed into XL-10 Gold cells resulting in a repaired vector. In each of the repaired vectors, a stop codon and NotI site was introduced at the +1 position of the codon encoding for TRPA1^1008^ by site-directed mutagenesis. Each of these plasmids were digested with NotI restriction enzyme followed by gel purifications. The linearized vector segments were then transformed into XL-10 Gold cells resulting in the final repaired vector. 3xFLAG-K989S/R996S/K997S/K1001S (4S) and K989S/R996S/K997S/K1001S/W1103A (4S and W1103A) hTRPA1 were generated by site-directed mutagenesis from the WT and W1103A hTRPA1 templates. W1103A DCTCaMBE and 4S TRP CaMBD peptides were similarly generated by site-directed mutagenesis from the WT peptides. For expression in *X*. *laevis* oocytes, 3xFLAG-hTRPA1 variant genes were subcloned into the combined mammalian/oocyte expression vector pMO (obtained from David Julius’ lab) at XbaI by InFusion cloning (Takara) prior to generating circular RNAs (cRNAs). eGFP-hTRPA1^971-1008^ was generated by first introducing hTRPA1^971-1008^ into pEG BacMam N-terminal His8 eGFP at the NotI site by InFusion cloning. eGFP-hTRPA1^971-1008^ was then transferred to pETDuetI at the BamHI site by InFusion cloning. 8xHis-eGFP-hTRPA1^1089-1119^ was similarly generated by InFusion cloning at the NotI site into into pEG BacMam N-terminal His8 eGFP. 8xHis-eGFP-hTRPA1^1089-1119^ was subcloned into the pMSP2N2 bacterial expression vector at NcoI/NotI by InFusion cloning. FLAG-6xHis-WT CaM, CaM_12_, and CaM_1234_ were subcloned into pRSF Duet-1at NdeI/XhoI by InFusion cloning. FLAG-6xHis-CaM_34_ in pRSF Duet-1 was generated by site-directed mutagenesis from the WT template. All DNA primers were ordered from Thermo Fisher Scientific, and all constructs were sequence verified using the Yale School of Medicine Keck DNA Sequencing Facility. Additional cloning details including primers used are provided in the accompanying Source Data file.

### Mammalian cell culture and protein expression

Human embryonic kidney cells (HEK293T, ATCC CRL-3216) were cultured in Dulbecco’s modified Eagle’s medium (Invitrogen) supplemented with 10% calf serum and 1x Penicillin Streptomycin (Invitrogen) at 37 °C and 5% CO_2_. Cells were grown to ∼85 to 95% confluence before splitting for experiments or propagation. HEK293T cells cultured to ∼95% confluence were seeded at 1:10 or 1:20 dilutions into 6- or 12-well plates (Corning), respectively. After 1 to 5 h recovery, cells were transiently transfected with 1 to 2 μg plasmid using jetPRIME (Polyplus) according to manufacturer protocols. The HEK293T cells used in this study tested negative for *mycoplasma* contamination.

### Bacterial protein expression and purification

Bacterial expression vector encoding 8xHis-eGFP-hTRPA1^1089-1119^ was transformed into BL21(DE3) cells and plated on a kanamycin (Kan) LB agar plate. An individual colony was selected to inoculate LB Kan overnight starter cultures at 37 °C from which 10 ml were used to inoculate 1 L of Terrific Broth Kan. Once the inoculated Terrific Broth Kan reached an absorbance (A) at 600 nm of 0.6, protein expression was induced using 1 mM IPTG and the temperature was dropped to 18 °C. After overnight induction, bacteria were harvested, snap-frozen with liquid nitrogen, and stored in a −80 °C freezer. Bacteria were cultured at 120 RPM for each step. For coproduction of eGFP-hTRPA1^971-1008^ and the suite of FLAG-6xHis-CaM variants, bacterial expression vectors were cotransformed into BL21(DE3) cells and plated on LB agar plates containing Kan and carbenicillin. Expression details are as above except that growth medias contained Kan and carbenicillin.

Bacterial pellets were thawed at 4 °C and resuspended in bacterial lysis buffer (50 mM Tris pH 8.0, 500 mM NaCl, 2 mM CaCl_2_, 5 mM β-ME, 1 mM PMSF, 10% glycerol, and 5 mg bovine DNase I). The lysis buffer additionally contained 60 mM imidazole when purifying the 8xHis-eGFP-hTRPA1^1089-1119^ peptide. Bacteria were lysed on ice using a Fisherbrand Model 120 Sonic Dismembrator (Thermo Fisher Scientific). The sonic dismembrator was set to 50% amplitude and cycled between 1 min on and 1 min off for a total of 20 min. Bacterial debris were pelleted using an Eppendorf centrifuge 5810R that had been cooled to 4 °C at 3900 RPM for 30 min. All constructs contained either a 6xHis or 8xHis tag and were purified by gravity-flow IMAC using lysis buffer equilibrated HisPur Ni-NTA resin (Thermo Fisher Scientific). To remove weakly, nonspecifically bound proteins, the resin was washed with low-molar imidazole wash buffer (50 mM Tris pH 8.0, 150 mM NaCl, 5 mM ß-ME, 0.1 mM PMSF, and 20 mM imidazole). Bound protein was eluted in 2 ml fractions with elution buffer (50 mM Tris pH 8,0, 150 mM NaCl, 2 mM CaCl_2_, 5 mM β-ME, 0.1 mM PMSF, and 300 mM imidazole) and an A_280_ measurement was taken for each fraction using a NanoDrop One (Thermo Fisher Scientific). For 8xHis-eGFP-hTRPA1^1089-1119^, protein containing fractions were pooled and exchanged into storage buffer (50 mM Tris pH 8.0150 mM NaCl, 2 mM CaCl_2_, and 0.1 mM PMSF) overnight by dialysis and then further purified by size-exclusion chromatography on a Superdex 200 column pre-equilibrated with storage buffer. eGFP-hTRPA1^971-1008^ and the suite of FLAG-6xHis-CaM variants were analyzed by Western blot as detailed below.

### Structure prediction

We used AlphaFold2-multimer with default parameters and Amber relaxation to generate all models. Computation was performed on the Farnam cluster at Yale Center for Research Computing. WT human CaM (amino acids 1–149) was used as the sequence for CaM. For hTRPA1, the complete cytoplasmic C terminus (amino acids 971–1119) was used (Fig. S1). One molecule of each protein was used for the modeling. Sequences for Human CaM and TRPA1 were retrieved from UniProt with accession codes P0DP23 and O75762. After obtaining predictions from AlphaFold2-multimer, we used the model with the highest confidence, as judged by average pLDDT, for all further analysis.

### Ratiometric Ca^2+^ imaging

Sixteen to twenty-four hours posttransfection, HEK293T cells were plated into isolated silicone wells (Sigma) on poly-L-lysine (Sigma)-coated cover glass (Thermo Fisher Scientific). Remaining cells were lysed for anti-FLAG immunoblotting to ensure equivalent expression, as detailed below. After adhering to the glass slide for 1 h, cells were loaded with 10 μg/ml Fura 2-AM (ION Biosciences) in physiological Ringer’s solution (in mM: 120 NaCl, 5 KCl, 2 CaCl_2_, 25 NaHCO_3_, 1 MgCl_2_, 5.5 Hepes, 1 D-glucose, pH 7.4; Boston BioProducts) with 0.025% Pluronic F-127 (Sigma) and 1% dimethyl sulfoxide, and incubated for 1 h at room temperature, and then rinsed twice with Ringer’s solution. Ratiometric Ca^2+^ imaging was performed using a Zeiss Axio Observer seven inverted microscope with a Hamamatsu Flash sCMOS camera at 20x objective. Dual images (340 and 380 nm excitation, 510 nm emission) were collected and pseudocolor ratiometric images were monitored during the experiment (Molecular Devices MetaFluor software). After stimulation with 100 micromolar AITC, cells were observed for 45 to 100 s. AITC was purchased from Sigma and was freshly prepared as a stock at 4x the desired concentration in 1% dimethyl sulfoxide and Ringer’s solution. Five microliters of 4x agonist was added to wells containing 15 μl Ringer’s solution to give the final 1x desired concentration. For all experiments, a minimum of 60 to 90 cells were selected per condition per technical replicate for ratiometric fluorescence quantification in MetaFluor with five biological replicates per experiment. Background signal was quantified from unresponsive cells and subtracted from quantified cells for normalization.

### Cell lysis and pull-down experiments

#### Whole-cell lysates—calcium imaging experiments

Resuspended HEK293T cells were pelleted at 1000 RPM for 10 min at room temperature. Media was removed, the cells were washed once with 1x PBS (Ca^2+^ and magnesium free, Boston Bioproducts) and lysed in 75 to 150 μl of TRPA1 lysis buffer (40 mM Tris pH 8.0, 150 mM NaCl, 5 mM DDM, 500 μM EGTA, EDTA-free cOmplete protease cocktail inhibitor tablet) at 4 °C while gently nutating. Cell debris were pelleted from the resulting lysates by centrifugation at 15,000 RPM for 10 min at 4 °C. Total protein concentration in lysates was quantified using a bicinchoninic acid) assay (Pierce). Equal concentrations of protein lysate were analyzed to assess protein expression as outlined below.

#### Whole-cell lysates—*X*. *laevis* oocytes

Individual *X*. *laevis* oocytes from TEVC experiments were lysed in 100 μl of a 1:1 mixture of TRPA1 lysis buffer and 6x reducing loading dye (Boston Bioproducts) by mechanical disruption and incubating on ice for 10 min. Lysates were clarified at 15,000 RPM for 5 min at room temperature and 5 to 10 μl lysates were analyzed to assess protein expression as outlined below.

#### CaM-agarose and anti-FLAG pulldowns

Experiments were conducted as previously reported (21). Briefly, 16 to 24 h posttransfection, HEK293T cells were washed with 1x PBS and lysed in 75 to 150 μl of TRPA1 lysis buffer (40 mM Tris pH 8.0, 150 mM NaCl, 5 mM DDM, 500 μM EGTA, EDTA-free cOmplete protease cocktail inhibitor tablet) at 4 °C while gently nutating. Cell debris were pelleted from the resulting lysates by centrifugation at 15,000 RPM for 10 min at 4 °C. Total protein concentration in lysates was quantified using a bicinchoninic acid assay (Pierce). Equal concentrations of protein lysate (100 μg) from each experimental condition were added to resins as specified below. 10% of loaded protein amount was reserved as a whole-cell lysate loading control. To obtain buffers with 0.1, 0.5, 1, 10, 100, 250, 500, 1000, or 2000 μM free Ca^2+^, the base TRPA1 lysis buffer was supplemented with 455 μM, 493 μM, 497 μM, 510 μM, 600 μM, 750 μM, 1 mM, 1.5 mM, or 2.5 mM CaCl_2_ as calculated using Ca-EGTA calculator v1.3 using constants from Theo Schoenmakers’ Chelator on the MaxChelator website (54).

CaM-agarose resin (Sigma-Aldrich) was buffer equilibrated overnight at 4 °C with lysis buffer at the intended Ca^2+^ concentration. EZview red anti-FLAG M2 affinity resin (Sigma) was buffer equilibrated for 1 h at 4 °C with lysis buffer at the intended Ca^2+^ concentration. One hundred micrograms lysates were incubated with 10 μl of buffer-equilibrated CaM-agarose or anti-FLAG resin for 1 h at 4 °C with gentle nutation. Resin beads were pelleted at 1000 RPM, washed 3 to 5 times with lysis buffer of the appropriate Ca^2+^ concentration, repelleted, and bound proteins were eluted by incubating with calcium-free TRPA1 lysis buffer supplemented with 5 mM EGTA (Boston Bioproducts) or 125 μg/ml 3xFLAG peptide (Sigma) for 30 min at 4 °C with gentle nutation.

For the peptide competition assays in Figure 8, 100 μg lysates prepared with 2 mM Ca^2+^ lysis buffer were supplemented with 0, 40, or 80 μM purified 8xHis-eGFP-hTRPA1^1089-1119^ peptide and then incubated with CaM-agarose resin for 1 h at 4 °C.

#### Nickel-NTA pull-down experiments

eGFP-hTRPA1^971-1008^ and the suite of FLAG-6xHis-CaM variants were copurified with Nickel-NTA resin as detailed above. Twenty microliters of each eluate was incubated with 10 mM EGTA for 1 h at room temperature to chelate all Ca^2+^. Precipitates were pelleted at 15,000 RPM for 5 min at room temperature. The supernatants were collected; the pellets were washed once with storage buffer and then resuspended in 10 μl 6x reducing loading dye (Boston BioProducts). Lysates and eluates (Fig. 7, *C* and *D*), as well as supernatants and precipitates (Fig. S2), were analyzed by Western blot as detailed below.

### SDS-PAGE and immunoblot

Samples were combined with 6x reducing loading dye (Boston Bioproducts) and separated on precast 4 to 20% SDS-PAGE gels (Bio-Rad). Gels were transferred onto polyvinylidene difluoride membranes (Bio-Rad) by semi-dry transfer at 15V for 40 min. Blots were blocked in 3% bovine serum albumin or 5% nonfat milk prior to antibody probing. The following primary antibodies were used in 1x phosphate buffered saline with 0.05% tween-20 buffer (Boston Bioproducts): anti-MBP (mouse, 1:30,000, New England Biolabs), anti-FLAG (mouse, 1:30,000, Sigma), anti-GFP (mouse, 1:5000 in 5% milk, Takara), and anti-tubulin (mouse, 1:5000 in 3% bovine serum albumin, Sigma). Horseradish peroxidase-conjugated IgG secondary anti-mouse antibody was used as needed (rabbit, 1:50,000, Invitrogen). Membranes were developed using Clarity Western ECL substrate (Bio-Rad) and imaged using a ChemiDoc Imaging System (Bio-Rad). Densitometric quantifications were performed with ImageJ software (https://imagej.net/ij/download.html). All quantified band intensities for eluted samples were divided by their tubulin-normalized input band intensities. These quantifications were further normalized to the maximum binding event within each biological replicate as indicated in figure legends. All pull-down experiments were performed for a minimum of three biological replicates as indicated in the figure legends. All uncropped blots are presented in Figures S3–S7. Antibody dilutions used were as previously reported (21, 46) and specificity was further established by (1) clean band signal only or primarily at expected sizes in the imaged blots (Figs. S3–S7) and (2) no signal in expected negative control lanes in pull-down experiments (*e*.*g*., Fig. 2, *C* and *D* no Ca^2+^, 4A, 4C, and 4E no Ca^2+^, 7A no Ca^2+^, 7C CaM_1234_, and 8C no Ca^2+^).

### Oocyte electrophysiology

Experiments were conducted as previously reported (21, 46). pMO vectors carrying 3xFLAG-tagged hTRPA1 constructs were linearized with PmeI. cRNAs were generated by *in vitro* transcription with the mMessage mMachine T7 transcription kit (Thermo Fisher Scientific) according to the manufacturer’s protocol and were purified with a RNeasy kit (Qiagen). cRNA transcripts were microinjected into surgically extracted *X*. *laevis* oocytes (extracted oocytes purchased from Ecocyte) with a Nanoject III (Harvard Apparatus). Oocytes were injected with 0.25 ng (WT hTRPA1), 0.1 ng (4S hTRPA1), or 0.2 ng (W1103A or 4S and W1103A hTRPA1) of cRNA per cell, and whole-cell currents were measured 24 h postinjection using TEVC electrophysiology. Currents were measured using an OC-725D amplifier (Warner Instruments) delivering a ramp protocol from −100 mV to +100 mV applied every second. Microelectrodes were pulled from borosilicate glass capillary tubes and filled with 3 M KCl. Microelectrode resistances of 0.7 to 1.2 MΩ were used for all experiments. Bath solution contained (in mM) 93.5 NaCl, 2 KCl, 2 MgCl_2_, 0.1 BaCl_2_, and 5 Hepes (pH 7.5). For experiments in the presence of Ca^2+^, BaCl_2_ was replaced with 1.8 mM CaCl_2_. Data were subsequently analyzed using pClamp11 software (Molecular Devices; https://support.moleculardevices.com/s/article/Axon-pCLAMP-11-Electrophysiology-Data-Acquisition-Analysis-Software-Download-Page). Oocytes were individually collected after recordings, lysed in 100 μl TRPA1 lysis buffer, and subjected to anti-FLAG and anti-tubulin immunoblot analysis to confirm construct expression as detailed above. All TEVC experiments were performed with a minimum of six oocytes per condition as indicated in the figure legends.

### Statistical analysis

All data quantification was performed in Microsoft Excel (www.microsoft.com). Quantified data presentation and statistical analyses were performed in GraphPad Prism (www.graphpad.com). Criterion for statistical significance for all tests was *p* < 0.05. The statistical tests applied, *p* values, and the number of biological replicates is indicated in the figures and/or the figure legends. For experiments where data are trending in a direction, yet not significant, the *p* values are indicated in the figures.

## Data availability

The data that support the findings of this study are available as an accompanying Source Data file. Uncropped blots are provided in Figures S3–S7 in the accompanying Supporting Information document. The 6V9W (https://www.rcsb.org/structure/6v9w) and 6PQQ (https://www.rcsb.org/structure/6PQQ) PDB files were used in this study. Models were built with ChimeraX version 1.10.

## Supporting information

This article contains supporting information (22).

## Conflict of interest

The authors declare that they have no conflicts of interest with the contents of this article.
