# Supplementary material for: Two calmodulin binding elements contribute distinctly to TRPA1 calcium desensitization
Source: J Biol Chem. 2025 Dec 13;302(2):111044. doi: 10.1016/j.jbc.2025.111044 (PMC12805100; doi:10.1016/j.jbc.2025.111044)
Supplement: Supplementary Material 2 [file mmc2.pdf]

Two calmodulin binding elements contribute distinctly to TRPA1 calcium desensitization

Gregory Quevedo[1§], Kehinde M. Taiwo[1§], Justin H. Sanders[1], Glory A. Adekanye[1], and Candice E. Paulsen[1,2]*

[1]Department of Molecular Biophysics and Biochemistry, Yale University, New Haven, Connecticut, USA.
[2]Wu Tsai Institute, Yale University, New Haven, Connecticut, USA.

[§]These authors contributed equally

*Correspondence: candice.paulsen@yale.edu

- Supporting Information Figures

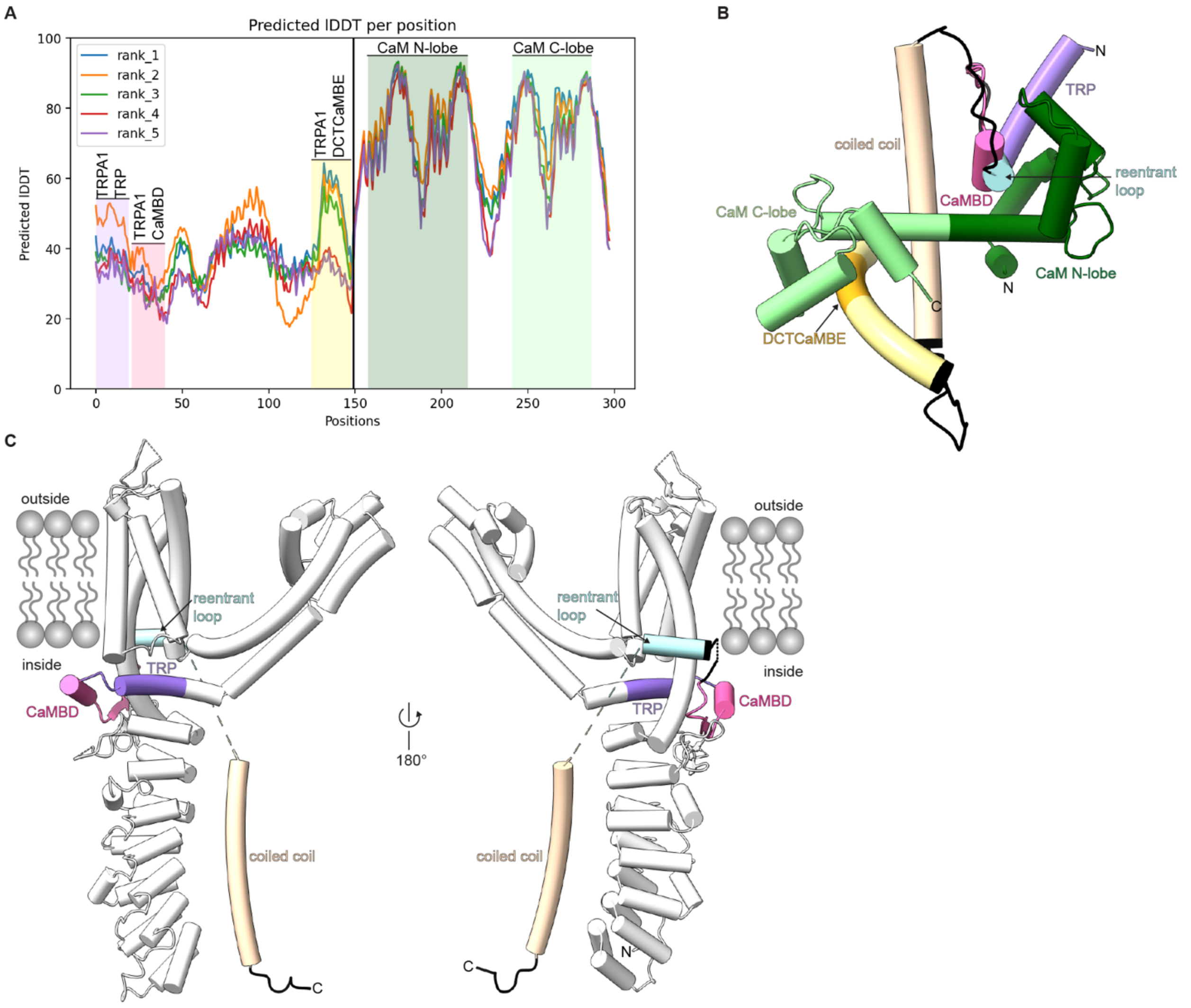

Figure S1. Statistics and analysis of AlphaFold2-Multimer model. (**A**) Confidence measurement (pLDDT plot) of AlphaFold2-Multimer model for hTRPA1$^{971-1119}$ docked to WT hCaM. The top five generated models are plotted. The rank 1 model was used for Figure 3C-E and 10. (**B**) Ribbon diagram of hCaM atomic model (green) in complex with the hTRPA1$^{971-1119}$. The hTRPA1 TRP helix (purple, residues 976-990), the CaMBD (pink, residues 991-1010), the membrane reentrant loop (light blue, residues 1017-1025), the coiled coil (light orange, residues 1038-1072), and the TRPA1 disordered C-terminus (yellow, residues 1089-1119) including the DCTCaMBE (goldenrod, residues 1102-1111) as predicted by AlphaFold2-Multimer. All other modeled features are denoted in black. This model predicts the TRP CaMBD and the membrane reentrant loop engage the CaM N-lobe while the DCTCaMBE engages the CaM C-lobe. (**C**) Ribbon diagram of a single WT hTRPA1 subunit atomic model for residues K447-E1079 (PDB: 6PQQ) is shown in white. Structural elements included in the AlphaFold2-Multimer model are colored as in (B). The membrane reentrant loop is likely predicted to bind CaM in (B) due to its abundance of hydrophobic residues. This helix has only been resolved in TRPA1 structures from lipid nanodiscs where it inserts into the membrane [1].

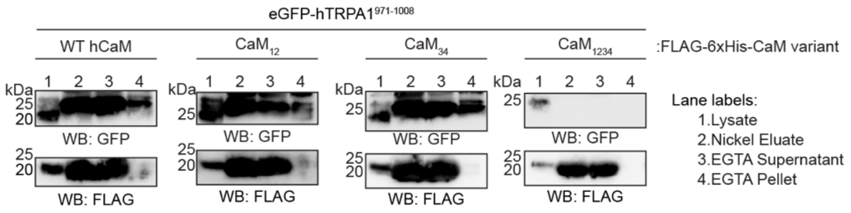

Figure S2. CaM lobe specificity and biochemical instability of TRP CaMBD. Immunoblotting analysis of GFP-TRP CaMBD (hTRPA1$^{971\text{-}1008}$) co-purified from *E. coli* with FLAG-6xHis-WT hCaM, CaM_{12}, CaM_{34}, or CaM_{1234} in the presence of 2 mM Ca^{2+}. Crude cell lysates (1), Nickel NTA eluates (2), 10 mM EGTA supernatants (3), and 10 mM EGTA precipitates (4) were probed using anti-GFP and anti-FLAG antibodies. Experiment is representative of 4 independent purifications.

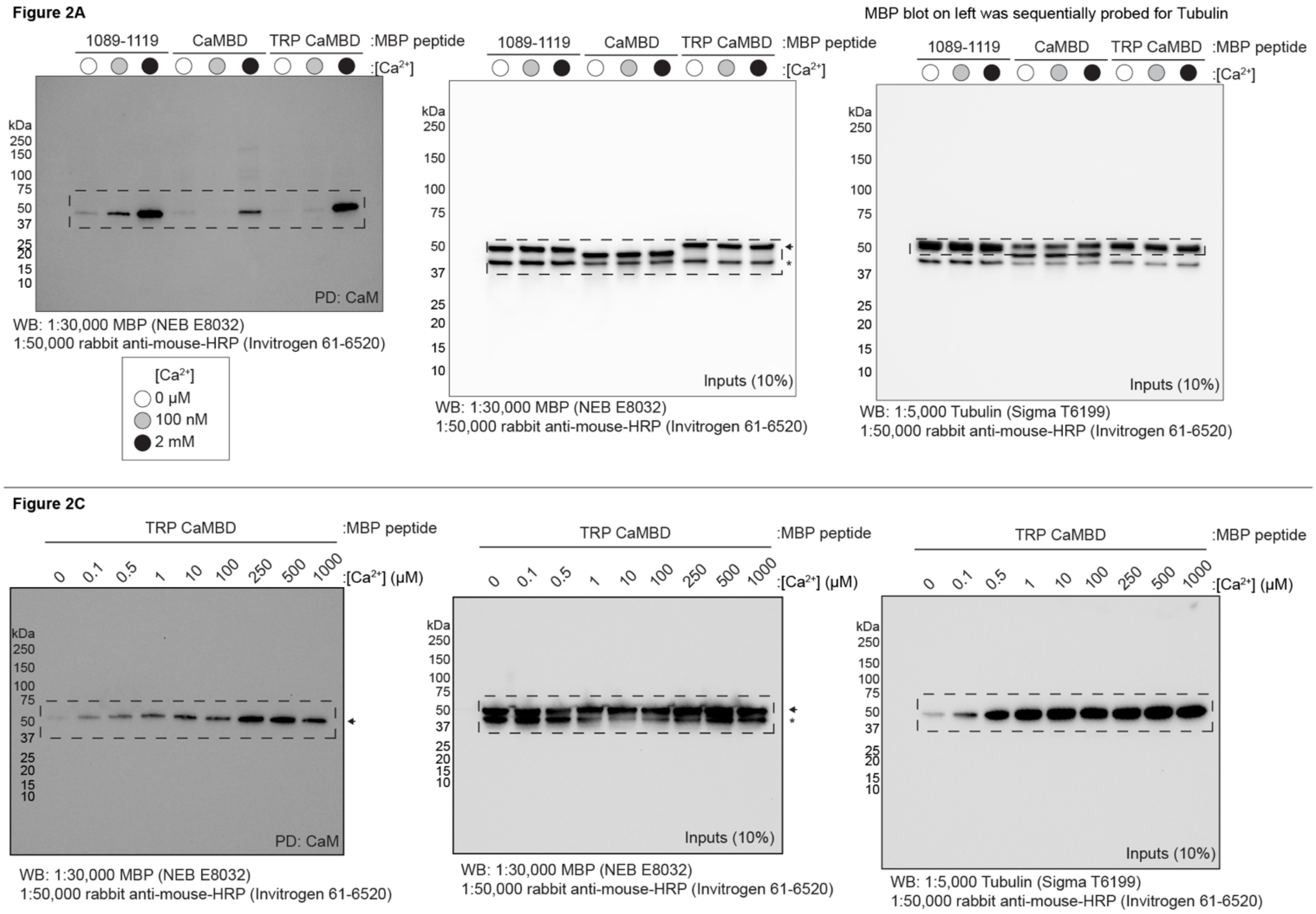

Figure S3. Uncut full Western blots shown in Figs. 2A and 2C. The regions surrounded by dashed lines represent the panels in the respective figures.

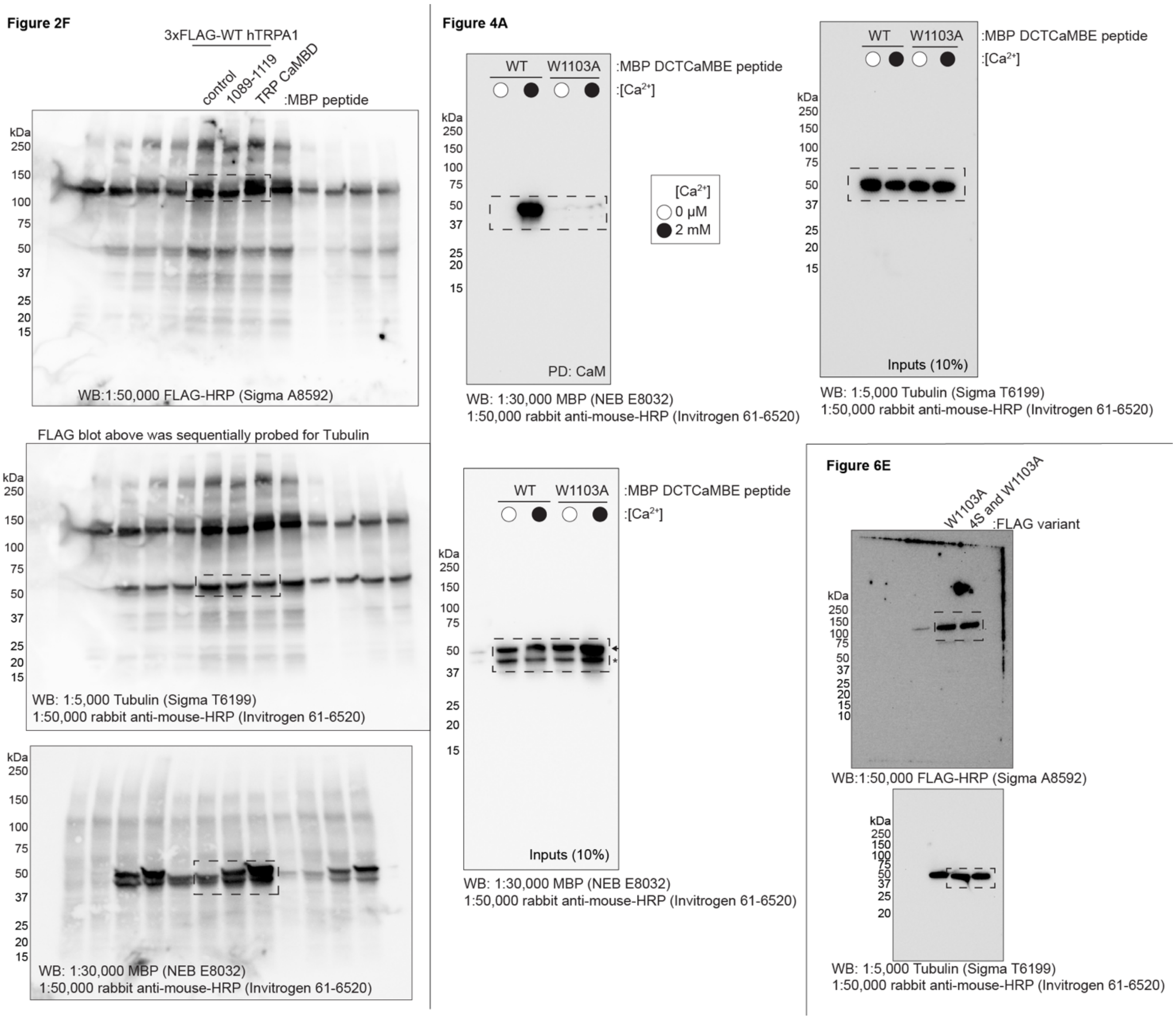

Figure S4. Uncut full Western blots shown in Figs. 2F, 4A, and 6E The regions surrounded by dashed lines represent the panels in the respective figures.

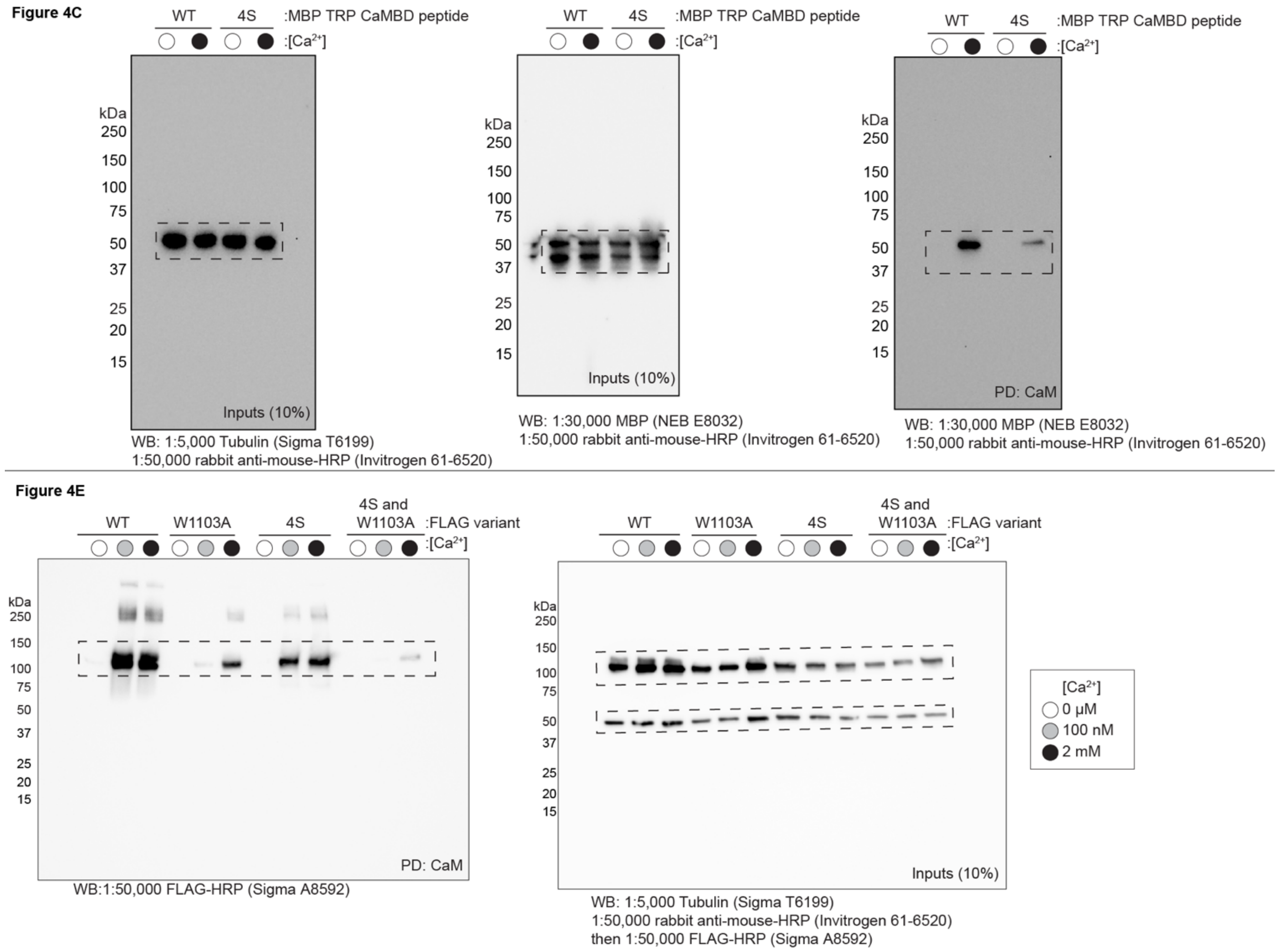

Figure S5. Uncut full Western blots shown in Figs. 4C and 4E. The regions surrounded by dashed lines represent the panels in the respective figures.

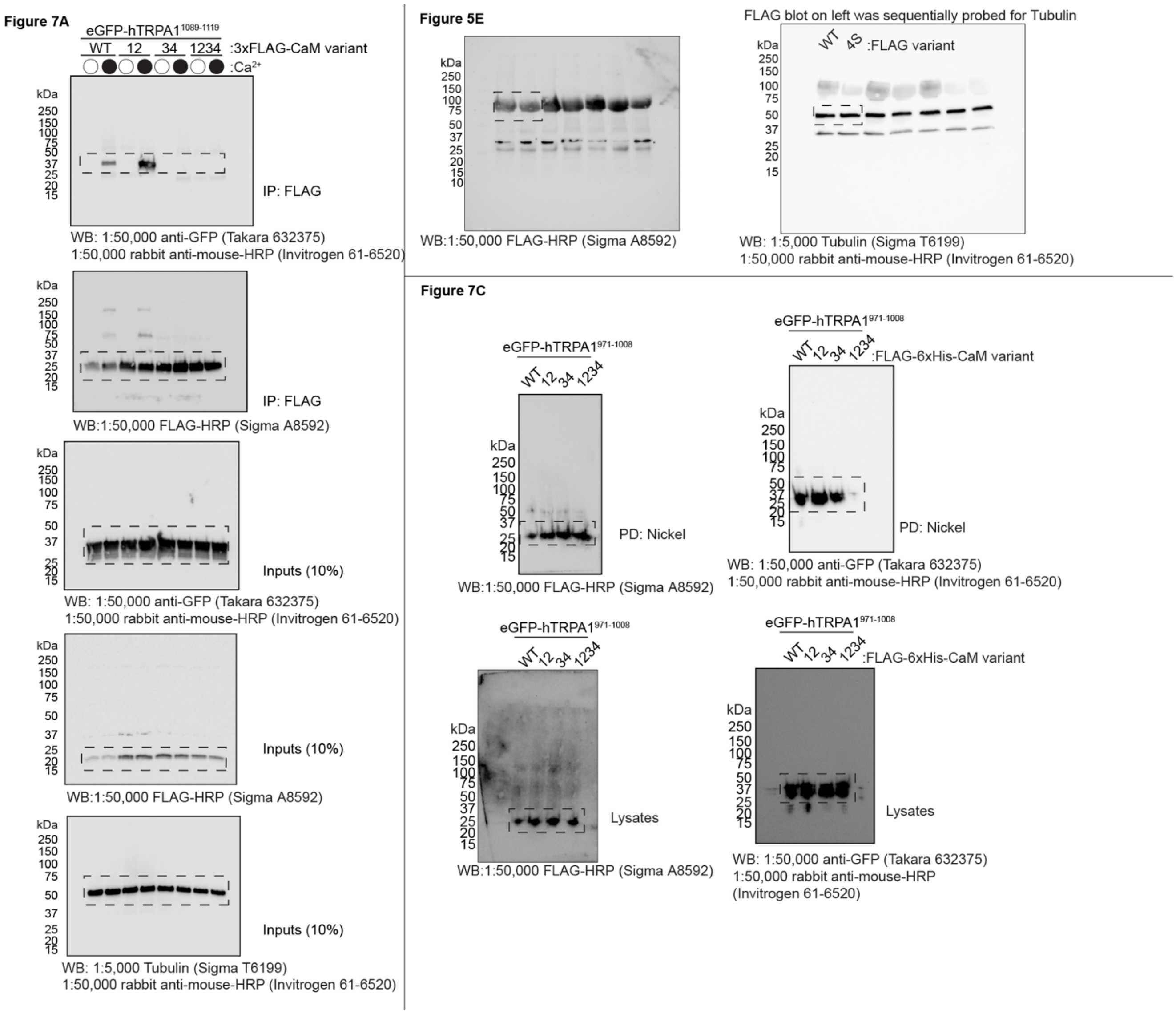

Figure S6. Uncut full Western blots shown in Figs. 7A, 5E, and 7C. The regions surrounded by dashed lines represent the panels in the respective figures.

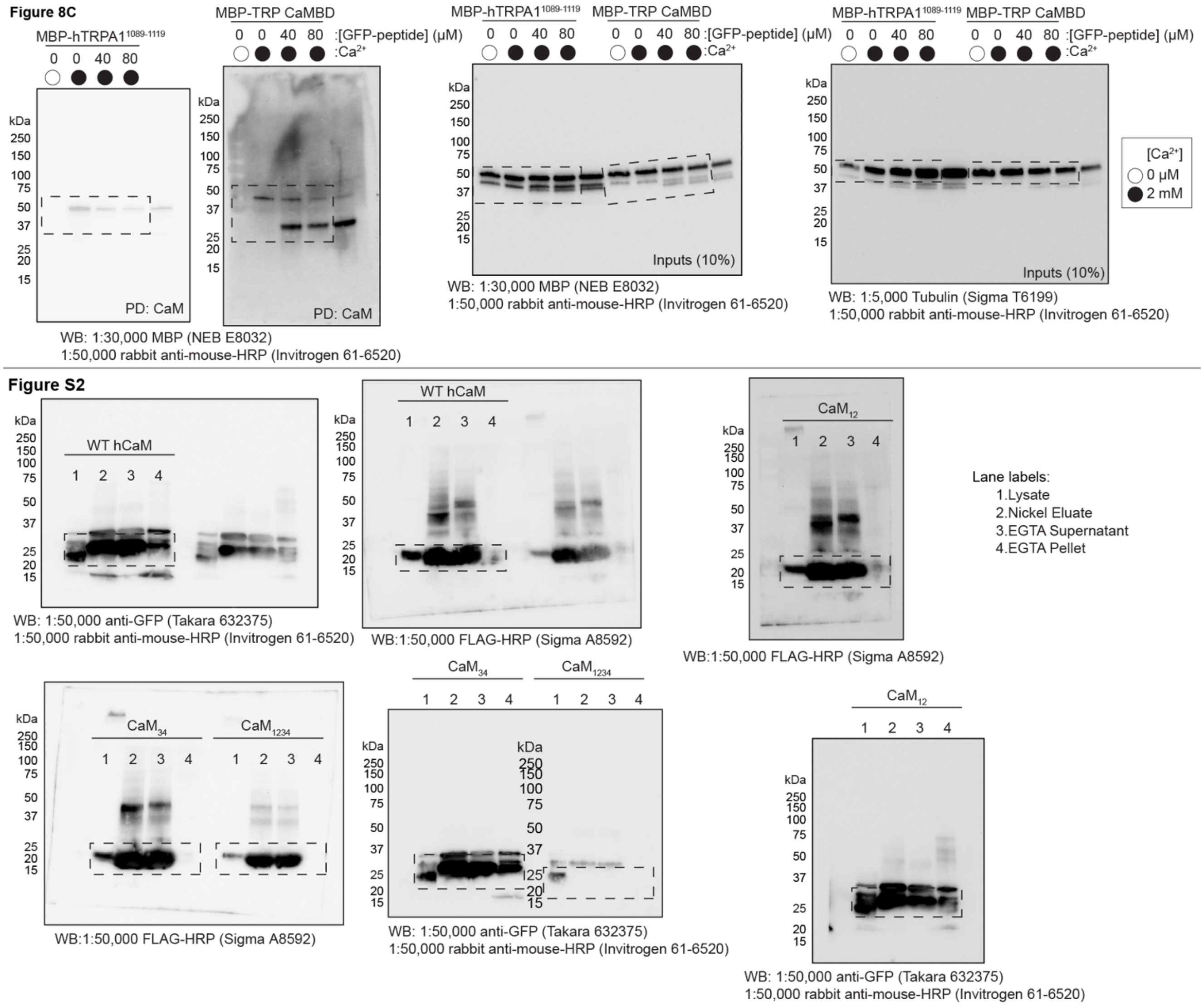

Figure S7. Uncut full Western blots shown in Fig. 8c and S2. The regions surrounded by dashed lines represent the panels in the respective figures.

SUPPORTING INFORMATION REFERENCES

1. Suo, Y.; Wang, Z.; Zubcevic, L.; Hsu, A. L.; He, Q.; Borgnia, M. J.; Ji, R. R.; Lee, S. Y., Structural Insights into Electrophile Irritant Sensing by the Human TRPA1 Channel. *Neuron* **2020,** *105* (5), 882-894 e5.
